# Using Information and Communication Technologies to Engage Citizens in Health System Governance in Burkina Faso: Protocol for Action Research

**DOI:** 10.2196/28780

**Published:** 2021-11-16

**Authors:** Sandrine Biau, Emmanuel Bonnet, Christian Dagenais, Manuela De Allegri, Zoumana Traoré, Abdoul Wahabo Ouedraogo, Abdramane Sow, Karina Dubois-Nguyen, Valéry Ridde

**Affiliations:** 1 Unité de santé internationale Université de Montréal Montreal, QC Canada; 2 AfricaSys Company Ouagadougou Burkina Faso; 3 UMR 215 PRODIG Institut de recherche pour le développement Aubervilliers France; 4 Department of Psychology Université de Montréal Montreal, QC Canada; 5 Heidelberg Institute of Global Health Heidelberg University Hospital and Medical Faculty Heidelberg University Heidelberg Germany; 6 Help – Hilfe zur Selbsthilfe Ouagadougou Burkina Faso; 7 Health and Development Working Group Action-Governance-Integration-Strengthening Ouagadougou Burkina Faso; 8 Centre Population et Développement Institut de recherche pour le développement, institut national de la santé et de la recherche médicale Université de Paris Paris France; 9 Institut de Santé et Développement Dakar Senegal

**Keywords:** health governance, ICTs, citizen participation, responsiveness, social responsibility, Burkina Faso, technology platforms, democracy, health systems, equity, West Africa, public health, participation, health policy

## Abstract

**Background:**

Health systems are complex systems involving a vast range of actors. In West Africa, they are often not accessible or responsive. Burkina Faso has widely expressed, in its public health policy, the need to improve both access to quality care and health system responsiveness. There is also a strong wish to give more voice to citizens. To support Burkinabè institutions in achieving these goals, we have developed an action research (AR) protocol.

**Objective:**

This paper presents the protocol that will address citizens’ participation in health policies and their empowerment through the expression of opinions, for accountability, as well as the strengthening of the health system using information and communication technologies (ICTs).

**Methods:**

Our approach will consist of (1) enabling people to express their opinions on the health system by means of a toll-free (TF) service coupled with an interactive voice server (IVS); (2) building an information base with anonymous and reliable data; and (3) conducting information awareness-raising activities, including knowledge transfer (KT) and advocacy, social integration activities, development of OpenData platforms, and the capitalization and media coverage of governance issues. For this purpose, the AR project will be implemented in Burkina Faso. The design uses a concurrent mixed-methods approach. This AR project will evaluate the acceptability, process, effectiveness, and economic costs of the device’s implementation. We will also analyze the potential for the data collected by the device to be used to improve practices.

**Results:**

Data collection is in progress; the TF number was officially launched on July 1, 2020, and data collection is planned to continue throughout 2021. By using mixed methods, our AR will be approached from a variety of perspectives. Mixed methods will support us in combining the partial insights into sophisticated realities from qualitative inquiries with the data analyses produced by quantitative research.

**Conclusions:**

This AR is expected to add knowledge on how to increase the empowerment of the population, especially the most vulnerable, to participate in democratic processes and enjoy and exercise their human rights. This protocol recommends implementing a low-cost, contextually adapted technology, associated with an evidence-based approach and carried out on a significant scale. The originality of this approach lies in the fact that it introduces a real AR dimension with local communities and nongovernmental organizations (NGOs), combined with an integrated strategy of KT and application throughout the project for all stakeholders.

**International Registered Report Identifier (IRRID):**

DERR1-10.2196/28780

## Introduction

Health is at the heart of the Sustainable Development Goals (SDGs). Indeed, a healthy society is essential for economic development, and in turn, economic development is essential to improve population health. To achieve economic development and a healthy society, health systems must be more responsive to people’s needs, as proposed by the World Health Organization (WHO) in 2000 [1,2]. WHO presents two main subdivisions of responsiveness: (1) respect for the person (dignity, confidentiality, autonomy of individuals and families regarding decisions related to their health) and (2) dignity for the patient (timeliness of care, access to social support networks during care, good quality of environment, choice of providers). For Mirzoev and Kane [3], this concept of responsiveness is complex and still underexplored [4,5]: “This perhaps explains [the] lack of comprehensive frameworks that go beyond the normative characteristics of responsiveness of health services and also justifies the examination of responsiveness as a distinct phenomenon” [3]. Successful responsiveness would be based on first clarifying people’s expectations with a view to supporting health system actors’ ability to respond appropriately to these expectations. This is an essential objective to achieve universal health coverage (UHC). Good governance with health system accountability is a critical component in UHC and quality of care [6]. Health system governance is a relatively modern concept that is not yet well assessed by research [4]. As a result, there is a lack of evidence on governance at the sub- and national levels. Nevertheless, by improving their governance, several low- and middle-income countries (LMICs) have made significant advances in ensuring access to quality health care compared to other states with similar wealth levels [7]. Despite the diversity of definitions of this concept, they all emphasize the importance of community participation and accountability [4,8]. Several studies have shown the important contribution of community participation to the success and sustainability of health programs and interventions [9,10]. It is considered an integral part of democracy and, as such, is recognized as an essential tool for health system governance [9]. However, beyond being a simple tool to improve governance, citizen participation is a fundamental right and an important objective to be pursued [11]. Unfortunately, in several sub-Saharan African countries, health systems are struggling to exploit the full potential of community participation [12], while community participation and accountability can be improved by providing effective channels for citizens to participate in decision making [13]. Indeed, putting in place institutional procedures for receiving complaints from users and health workers and disseminating basic knowledge about patients’ rights are two ways to improve governance and strengthen the health system [14].

Giving users a voice would promote health workers’ accountability to patients in order to positively influence their behavior and thereby improve care quality [15,16]. However, health workers’ voices must also be included because of their involvement in the system [17,18]. Their job satisfaction is an essential parameter that influences productivity as well as the quality of work [19,20]. Job satisfaction has a significant impact on quality, efficiency, and commitment to work [21] and, at the same time, on health care costs [22]. It is crucial that workers’ voices be heard, both to gain a deeper understanding of the health care system’s dysfunctions and to better assess the potential impact on patient satisfaction [23].

Various methods exist for collecting complaints, each with its own limitations: suggestion boxes, which require knowing how to write; satisfaction surveys, which are sporadic and not proactive; recourse to justice, which is too often administratively cumbersome and costly; or complaint services, which raise issues of discretion with users and health workers [24]. Too many people are afraid of speaking up or complaining or are deterred by the lack of reaction to problems on the part of the system actors and the government [6].

Information and communication technologies (ICTs), such as mobile phones, offer new opportunities in the health field. In fact, recent years have seen a drive among international and local agencies, governments, and institutions to develop projects using ICTs. Access to mobile technology has become more democratic [25], such that the population can now become actors in information production [26]. Mobile health (mHealth) has the potential to support health systems [27] and help fill persistent gaps by facilitating communication and information exchange [28]. In Africa, there are many such interventions because of the approach’s many advantages (access in hostile areas, high mobile phone penetration rate, lower costs, ease of use, etc) [29,30]. Many interventions involve using the short message service (SMS) [31,32] or voice messages via toll-free (TF) numbers [33,34] for purposes of information sharing or prevention.

In Burkina Faso, in 2014 and 2015, a pilot action research (AR) project to evaluate the technical, social, and instrumental relevance of a TF call service with an interactive voice server (IVS) was tested in 11 sectors in Ouagadougou to give the population and health workers a voice to express their opinions on their health system (Multimedia Appendix 1). That AR project showed that a TF number coupled with an IVS is a solution suited to the Burkinabè context for the free expression of views on the health system [35]. Our aim now is to scale up this AR project over a wider region than the pilot project, particularly in rural, semiurban, and urban areas. This will make it possible to compare results according to residential environment and sociological conditions (cultural differences between regions).

The AR protocol for setting up a TF-IVS presented here proposes to deploy a technological platform and strengthen the capacities of local implementing partners in Burkina Faso to improve citizen engagement and make health systems more responsive to their needs.

## Methods

### AR Approach

We have chosen an AR approach. The researchers involved have perfect mastery of this concept, which is adapted to both the country’s context and the problem [36]. The AR approach will make it possible to continuously adapt the project and ensure maximum effectiveness for better citizen participation and consideration of users’ needs in health decision making. AR is “a participatory democratic process that involves the development of practical knowledge of interest to the population, rooted in a participatory worldview that emerges at this particular historical period” [37].

Using an AR approach, which enables rapid learning, this project (named TechnOlogie PartIcipation Citoyenne en santé [TOPICs]) will study the acceptability, process, effectiveness, and financial cost of the digital device for citizen participation in health in urban and rural areas. It will also analyze the potential for using the evidence produced—in other words, the potential of the data collected by the device (TF-IVS) to improve practices. Finally, this research will draw lessons from the AR implementation to facilitate a possible national scale-up in the event of a successful outcome.

### General Objectives and Research Questions

The objective of this project is to evaluate, in Burkina Faso, the large-scale implementation and acceptability of a technological system for collecting people’s opinions on the health system, with a view to improving health governance and informing citizens about their health-related rights and duties.

The research questions (RQs) are as follows:

RQ1. What are the challenges involved in setting up, operating on a large scale, and using the digital device to gather the opinions of users and health workers about the Burkinabè health system?

RQ2. What factors internal or external to the AR project influence acceptance of the digital device to gather the opinions of users and health workers about the Burkinabè health system?

RQ3. What factors internal or external to the AR project influence the decision making of the actors or decision-making bodies based on the results of the digital device?

RQ4. How effective is the digital device in gathering the opinions of users and health workers about the Burkinabè health system?

RQ5. What are the economic costs of setting up and operating a system to collect the opinions of users and health workers on the Burkinabè health system?

RQ6. What are the effects of the knowledge transfer (KT) strategy and process on decision making?

### Evaluability Assessment

We first focused on defining needs and challenges, as well as on discussions with structures already carrying out similar experiences (to avoid duplication and ensure our process would be complementary to theirs).

We met with authorities and stakeholders from the central government, the region, and localities in the targeted areas. Then we collected information and made an inventory of the existing situation. This was followed by a process of validating our information and strategies. This work with stakeholders established the beginning of project ownership, a shared understanding of the implementation strategies, and mutual agreement on expected responsibilities and results.

**Figure 1:**
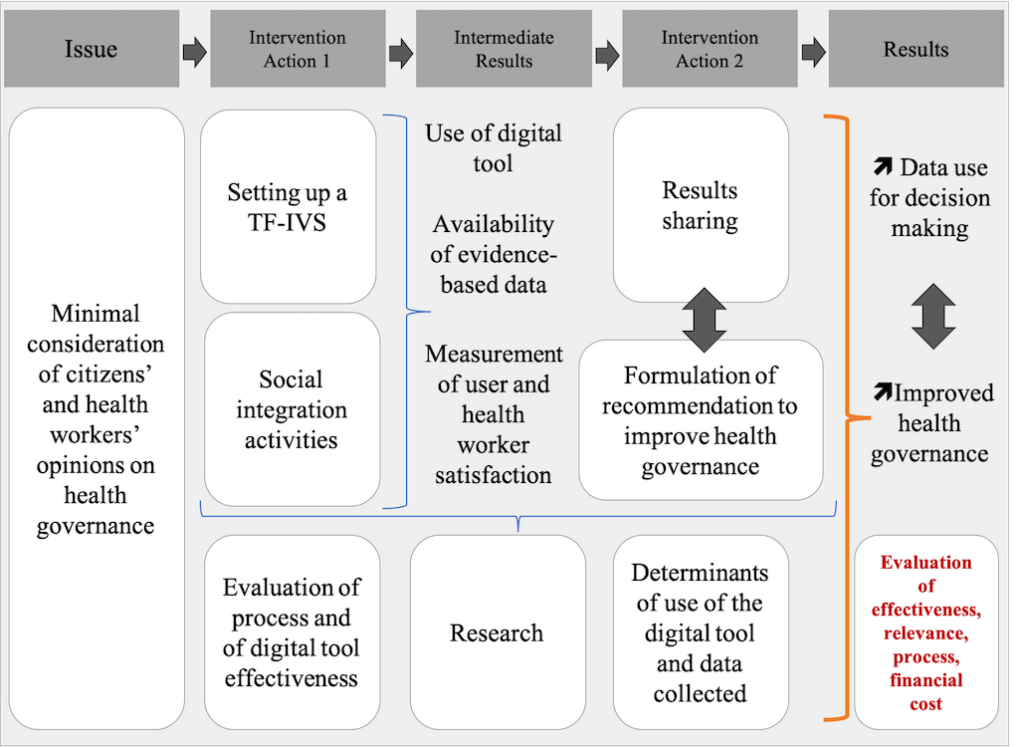
Theory of change. TF-IVS: toll-free service coupled with an interactive voice server.

Finally, we defined the geographic area to be covered by the AR project based on the possibility of applying our AR project both in urban areas (more suited to ICT implementation) and in rural areas in order to verify its feasibility. The issues related to project implementation in both rural and urban areas were among the main challenges considered in this preparation phase. Figure 1 presents the theory of change for the AR project.

### Types of Evaluation

Different types of evaluations will be carried out:

- Assessment of the relevance (instrumental, technical, contextual; RQ1, RQ2, and RQ4) of the digital device for expressing the opinions of the population and health workers about the Burkinabè health system
- Process evaluation to assess the factors (related to technology, context, support tools such as communication or training, etc) influencing the digital device’s implementation (RQ1-RQ3 and RQ6)
- Prospective cost analysis (RQ1-RQ5) to estimate the full economic costs associated with the AR project
- Evaluation of the digital device’s effectiveness for producing simple and evidence-based results to influence decision making needed to adapt the health system to the needs of the population and of health workers (RQ1-RQ6)

This AR project is based on evidence gathered through both a rigorous literature search and a previous pilot experiment that identified various subjects on which health center users and health professionals wanted to give their opinions on the health system [35].

Furthermore, because health interventions are strongly influenced by their context, this intervention was carefully conceptualized in relation to the Burkinabè situation [38]. As indicated by Craig et al [38], the following dimensions were taken into account: (1) the contexts in which the AR is intended to be applied, (2) the ways in which causal mechanisms and modes of delivery are context dependent, and (3) the dimensions of the context and how they are defined. As such, the Template for Intervention Description and Replication (TIDieR) for population health and policy (PHP) interventions will be helpful to report our AR project [39].

A group of four partners will implement this AR project: the International Health Unit (USI) of the University of Montreal, Canada; the Institut de recherche pour le développement (IRD), France; Heidelberg University, Germany; and the nongovernmental organization (NGO) Action-Gouvernance-Interaction-Renforcement, groupe de Santé en Développement (AGIR-SD), Burkina Faso. The AGIR-SD will conduct activities with the assistance of the IRD and University of Montreal researchers and the USI. The AGIR-SD is also involved in a teamwork process with a civil society organization (CSO) to raise public awareness and to organize information activities on the existence of the TF-IVS. Collaboration will be established between the German NGO Hilfe zur Selbsthilfe (HELP) and the AGIR-SD for coverage of the AR zones.

### Site Selection

The TF-IVS will be implemented on a larger scale than the pilot project developed in Burkina Faso in 2014 [35], particularly in rural, semiurban, and urban areas, in order to be able to compare results according to place of residence and contextual realities, while taking into account the project’s resources.

**Figure 2:**
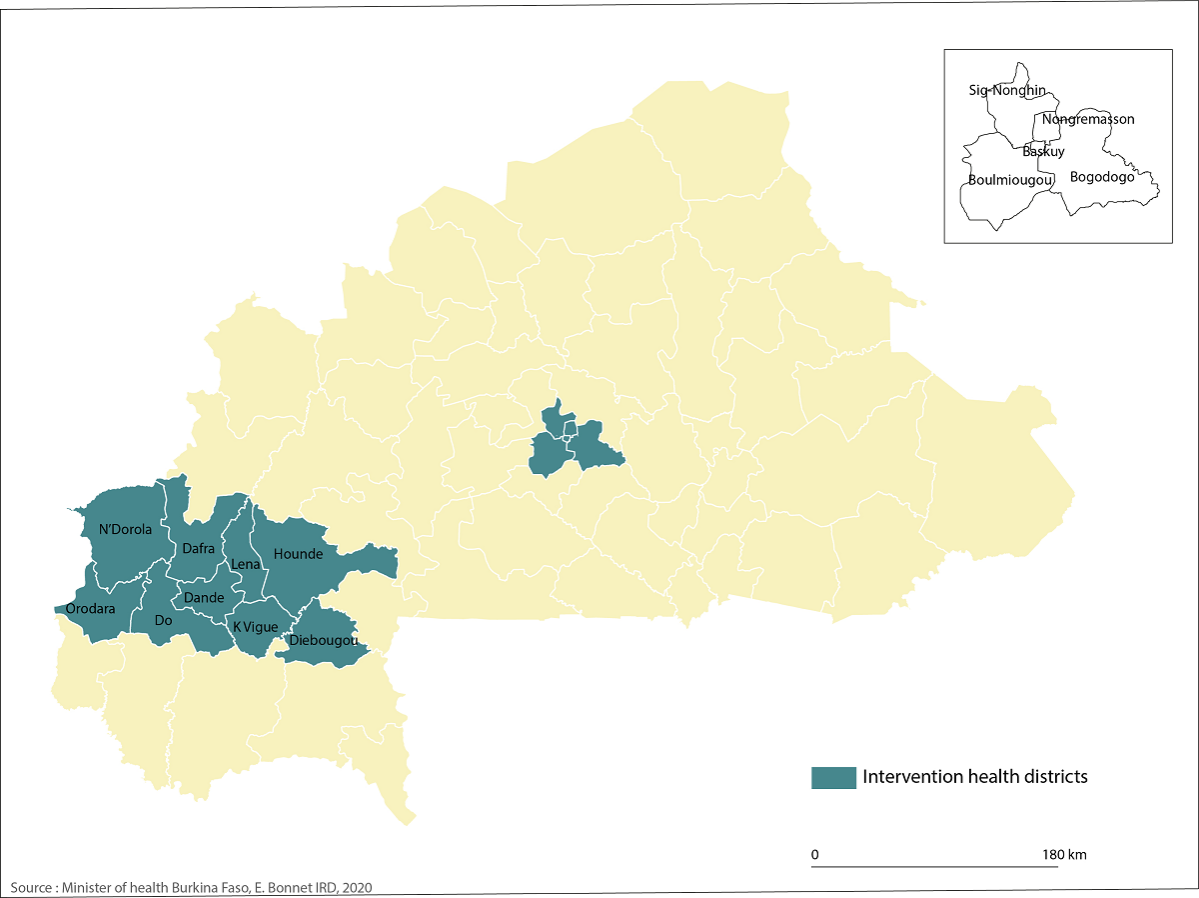
Health districts. IRD: Institut de recherche pour le développement.

Of the three regions targeted by the AR project, two include the country’s two largest cities (Ouagadougou and Bobo Dioulasso); these have a predominantly urban population. Diébougou is a rural area, which represents a challenge to test technological AR. The TF-IVS project will cover 14 health districts located in 3 regions (see Figure 2). It should be noted that in rural areas, just over half of the population (55.8%) owns a mobile phone, whereas in urban areas, almost 9 of 10 people (87.0%) own a mobile phone [40].

### Study Participants

The direct beneficiaries are the users of health services in the health regions of the Center, the Hauts bassins, and the province of Bougouriba and, more particularly, women, girls, and the poor, as well as health workers and community relays. Secondary beneficiaries are the entire population; health system managers; decision makers; and political, administrative, customary, religious, and CSOs.

### Study Setting and Population

In Burkina Faso, three operators share the mobile telephony market: Onatel, Telecel, and Orange. The mobile phone penetration rate was 95.95% in 2019 (Regulatory Authority for Electronic Communications and Posts [RAECP]) [41]. Mobile phone ownership rates are 79.4% for men and 51.7% for women. Almost two-thirds (64.3%) of Burkinabè aged 15 years or over owned a mobile phone in 2014. It is also known that half of the poorest 20% of individuals have a functional mobile phone, as do 82.3% of the richest 20% of individuals [40]. At the national level, 87.4% of individuals who did not own a phone but had used one in the past 30 days used the phone of a household member, and 12.4% used the phone of a non-household member. This access to phones is an opportunity for the initiative to succeed.

**Figure 3:**
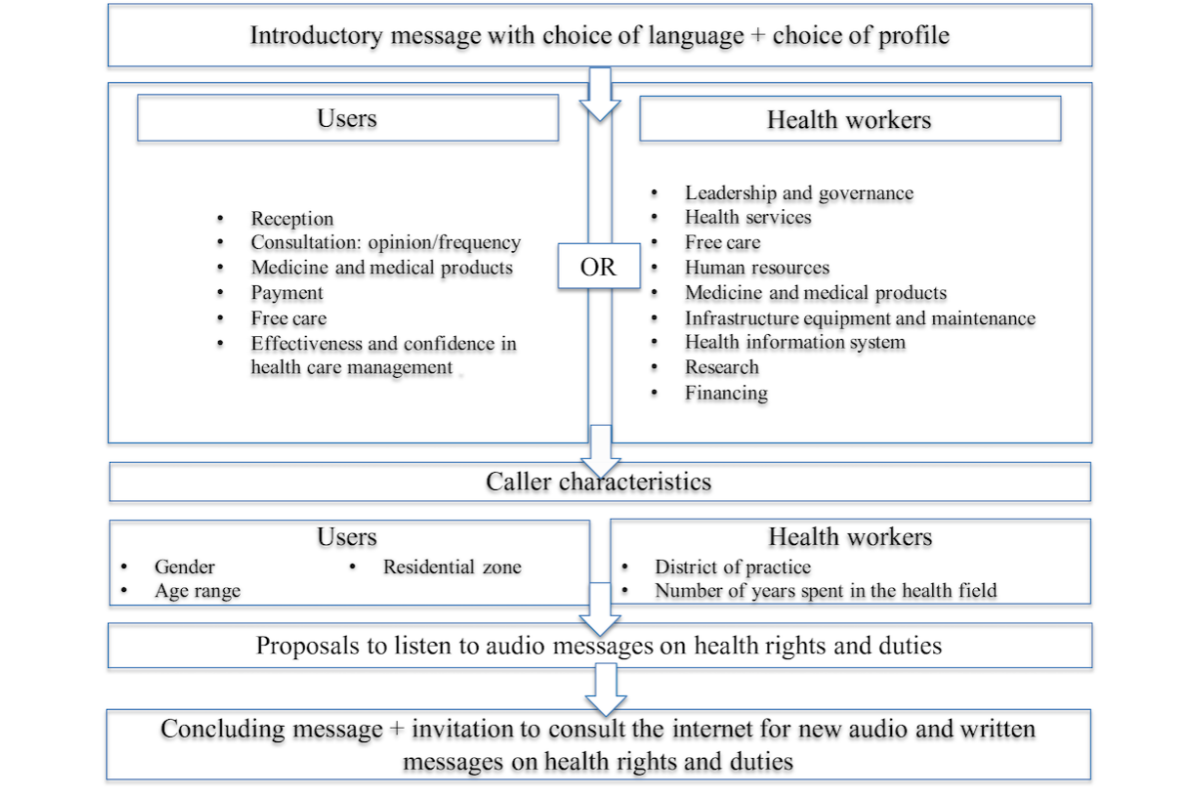
Diagram of surveys used.

This health AR project will develop a TF number, free of charge, reachable from any landline or mobile phone, anonymous and available 24 hours a day, 7 days a week, in any of the 9 main languages spoken in the areas targeted by the project. Phone users will use their phone keypad to respond to questions. Service users and health workers will answer two different questionnaires designed for their group. Figure 3 outlines the various phases of the call and the separate themes covered for service users and health workers.

While maintaining anonymity for ethical reasons, the system will request elements characterizing the caller, such as the city in which the health center is located, the health district in which the health worker is practicing, the sex of the caller, and their age group. The data collected will be stored on a local server secure and replicated in Burkina Faso. The data will be instantly transferred to the database accessible via the internet for collection.

Thus, persons calling the TF number will be able to answer the IVS questionnaire (Figure 3) and then will get an opportunity, at the end of the satisfaction measurement questionnaire, to receive information about their health-related rights and duties. The information about health rights that will be disseminated will have been validated by the project’s technical committee.

**Figure 4:**
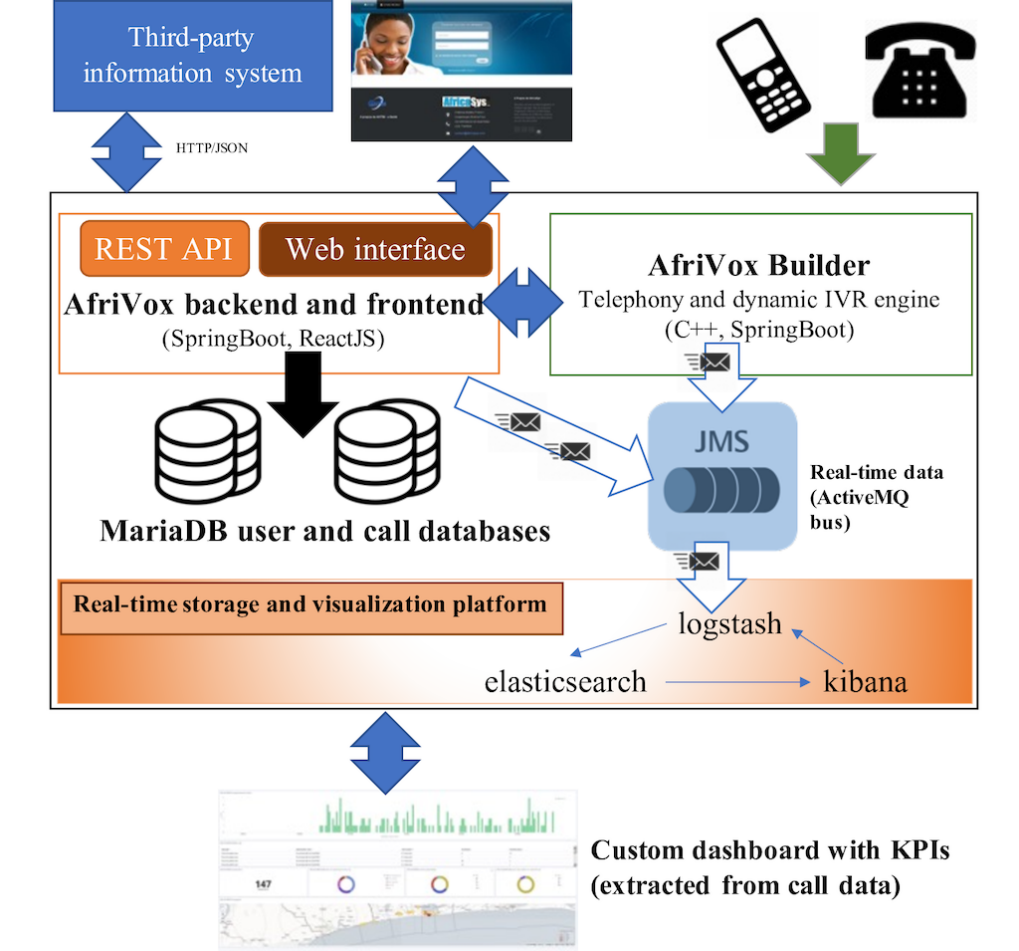
TF-IVS technological platform. ActiveMQ is free software implementing the JMS specification. C++ is a computer programming language. MariaDB is free relational database management software. ReactJS is a computer programming language of the graphical user interface (web). SpringBoot is a set of libraries for the Java computer programming language. JMS: Java Message Service; JSON: (IT) format for structured data exchange; KPI: key performance indicator; REST API: Representational State Transfer Application Programming Interface; TF-IVS: toll-free service coupled with an interactive voice server.

**Figure 5:**
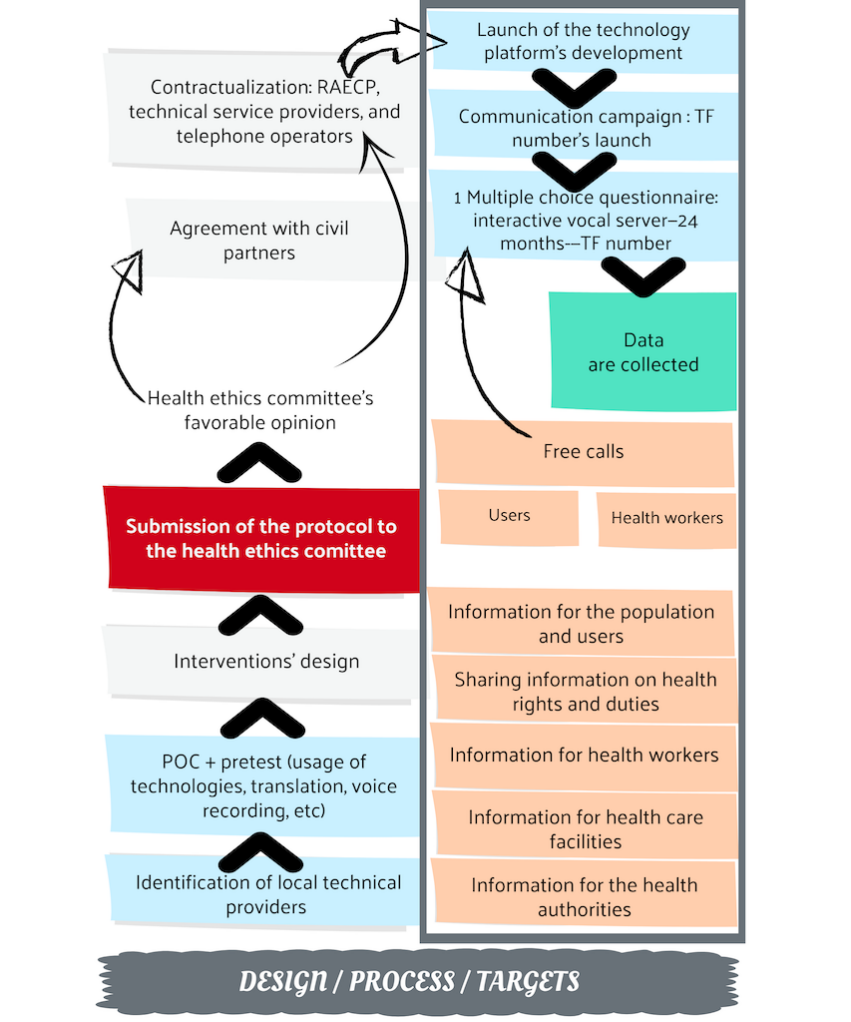
Design process and targets of the AR project. AR: action research; POC: proof of concept; RAECP: Regulatory Authority for Electronic Communications and Posts; TF: toll free.

A technological platform will be developed and managed by AfricaSys, which supported the pilot TF number project tested in 2015 [35] by the NGO AGIR-SD. The technological platform (Figure 4) will consist of a combination of the TF number, the IVS, the database, a computer, and a website (where the database is located). Figure 5 summarizes the design, process, and targets of the AR project.

The required infrastructure is based on continuous electrical, telephone, and internet access. A power generator is installed in Ouagadougou, in a facility belonging to the Institut de recherche au développement, to cope with frequent power and internet interruptions.

Support activities will be carried out. Communication activities will focus mainly on media and nonmedia communication campaigns in French and local languages. These will be implemented throughout the project to make known the device being made available to the population and health workers to express their opinions. Different communication channels will be used to enable the project to effectively achieve its communication objectives. This media campaign will be reinforced by social integration activities in the form of information and awareness-raising sessions in the community (talk-debate, discussion groups, sketches, etc). These will be aimed at encouraging people to use the digital devices available. Through a participatory approach, the project will involve several stakeholders and implement four packages of community activities:

- Contact with local, religious, and customary authorities for the project presentation and the negotiation of their adoption
- Meetings with different associations with common characteristics (people with disabilities, older and young people, women, craftspersons)
- Public awareness sessions for the general public
- Interactive radio broadcasts on topics related to rights and duties to health with the testimony of the population and based on data collected on satisfaction by the TF-IVS

### Target Size

The AR project will consider the population of the three areas targeted as well as the associated health workers. The total population covered in the 14 health districts covered by the TF-IVS is presented in Table 1 [42].

Table 2 presents the situation (in 2018) of the public and private health infrastructures and the health worker population in the TF-IVS coverage areas [43]. The following are considered health workers: specialist physicians, general physicians, pharmacists, dental surgeons, specialized nurses, biology laboratory technicians, state-registered nurses, registered nurses, state midwives, and state medical officers [44].

**Table 1:** Population covered by the AR^a^.

Location of the regional health department	Women (n)	Men (n)	Total (n)
Center	1,476,075	1,490,232	2,966,307
Hauts bassins	1,126,020	1,101,257	2,227,277
Diébougou	77,048	71,222	148,270
Total	2,679,143	2,662,711	5,341,854

^a^AR: action research.

**Table 2:** Public and private health infrastructure and health worker population in the AR^a^ areas in 2018.

Location of the regional health department	Public health infrastructure (n)	Private health infrastructure (n)	Health worker population (n)
Center	162	135	3515
Hauts bassins	208	24	1847
Diébougou	25	0	159

^a^AR: action research.

### Data Collection and Analysis

To carry out the different types of evaluations proposed before, we will adopt both a mixed-methods [45] and a multimethod approach.

#### Evaluation of the Implementation, Operation, Use, Acceptance, and Efficiency of the Digital Device (RQ1-RQ4)

We will approach these different evaluations from diverse angles, each of which is explained next, first from a quantitative and then from a qualitative standpoint. This subsection will address RQ1-RQ4 cited before.

A *quantitative approach* will be adopted to assess the device’s use and establish a typology of users. This will make it possible to analyze the evolution of the device’s use and the progression of user satisfaction (RQ1, RQ2, and RQ4).

##### Study Population and Sampling

The study population consists of all users of the digital device implemented. The data collected over the course of the AR project through the digital device will constitute data sources for quantitative analysis (usage evaluation, effectiveness evaluation, number of calls, number of responses from the digital device after each communication, etc). Thus, we will look at the data in the IVS dashboard. Spatial analyses will complement this quantitative component to assess whether there are geographical inequalities in the satisfaction with and use of the digital device. These analyses will also make it possible to evaluate the use of the TF number by location (geographical zone or health district). All the data will be organized in a geographic information system (GIS) using the QGIS software (Open Source Geospatial Foundation) program.

##### Collection Instruments

The quantitative data will be derived from the satisfaction questionnaire administered to people calling the IVS through the digital device set up by the AR project (see before). The questionnaire (Multimedia Appendix 2) has been constructed based on a review of the scientific literature and survey tools deployed to measure health system quality, services and care, perceived health status, and care seeking (use of and access to services). Several steps regarding the validation of the testing instrument were necessary. They include (1) validation of the questionnaires and messages on health rights and duties by the project team, the steering committee, and the communication unit of the Ministry of Health; (2) translation of the questionnaires and messages and validation of the translations (in terms of form and content); (3) validation of the tools by the ethics committee included in the research protocol; and (4) digitization of the tools in the IVS and testing the tool’s functionality (verification of the coherence between the questions and the question jumps, and the data’s synchronization) by the project team and the technical partner AfricaSys.

##### Data Analysis

Quantitative data will be subject to automated descriptive analysis and disaggregated by sociodemographic characteristics (gender, age groups, place of residence) and types of health facilities involved. The variables of interest will be those asking for the user’s opinion on the user’s care. We will conduct a descriptive evaluation to identify trends in caller characteristics. For all analyses, we plan to use R (Lucent Technologies) [46], an open source software package for computing and statistical graphs.

All data will be mapped at either the provincial level (health service users) or the health district level (health personnel). This will make it possible to identify possible territorial inequalities. Spatiotemporal statistics will be produced (Kulldorff spatial scan method [47,48]) to assess potential geographical fluctuations in time and territory (12 months) of calls and satisfaction variables.

Likewise, an analogous analysis will be carried out to identify associations between the use of digital devices by the population and actions to encourage their use. Indeed, we plan to document all communication and outreach activities daily throughout the implementation. This information will be used to measure the relationship between the number of communication campaigns, their intensity, their type, and the number of calls. The idea is also to measure the duration of the effect of the campaigns on the number of calls, that is, we will calculate how long after the last campaign the number of calls drops.

This analysis will be further developed through the qualitative component.

Demographic data (age group, gender, municipalities, type of health facility concerned) will provide details on the characteristics of individuals who have used the digital device. Comparing these data with those of the general population census of municipalities covered by the AR will provide an indication of profiles of users of the digital device. The numbers of proper completions of the questionnaire via the TF-IVS will be interpreted as an indicator to measure the ease of use of these digital devices. Nevertheless, selection biases may persist despite the cultural consideration and adaptation of the communication strategy in the field and will be considered in the data analysis.

A *qualitative approach* will also be adopted to determine the conditions for success or failure of the AR project as a whole and of the digital device and the various support activities specifically [49]. In particular, we will seek to identify factors related to the use of the digital device by the population, on the one hand, and those associated with the use of the project results by social and health authorities, on the other (RQ2 and RQ3). This will provide an understanding of the technical challenges of operating and using the digital device and using the results produced (RQ1). Similarly, we will conduct an evaluation of the project’s effectiveness as perceived by the various stakeholders (RQ4).

#### Acceptability Evaluation (RQ1-RQ3)

The success of an AR project’s implementation depends on its acceptability, both for the implementing actors (patients, researchers, health professionals) and for the beneficiaries (patients or health professionals) [50]. Although acceptability is a necessary but not sufficient condition for the effectiveness of an AR project [51], we believe it is crucial to study it, given the exploratory nature of the research and the technological innovation of this AR project. We have adapted the conceptual framework proposed by Sekhon et al [52], which characterizes acceptability as a multidimensional construct that illustrates the degree to which people who give or obtain a health care intervention consider it suitable, based on planned or experienced psychological and emotional responses to the intervention. The seven dimensions of acceptability proposed in this framework will be measured. These are (1) the emotional attitude of the targets (users and health workers) toward the AR, (2) the coherence/understanding of the AR, (3) the opportunity cost of participating in the AR, (4) the perceived effectiveness of the AR, (5) self-efficacy, (6) the perceived amount of effort required to take part in the AR, and (7) the degree to which the AR is a good fit with an individual’s value system.

The implementation study will be based on the Consolidated Framework for Implementation Research (CFIR; RQ1-RQ4) [53]. This conceptual framework recommends basing the study of factors influencing the implementation on five main dimensions: (1) the characteristics of the AR, (2) the external context of the health facilities, (3) the internal structure of the health facilities, (4) the characteristics of individuals, and (5) the process of implementing the AR project. Each of these dimensions includes useful constructs, chosen based on about 20 other frameworks. Given the exploratory nature of the AR project, we will not limit ourselves to these constructs during data collection and will endeavor to capture all additional information that may be useful to understand its implementation.

##### Sampling Population and Collection Instruments

Concerning RQ1-RQ4, three main targets will be of interest. These are:

- Target 1: project team members (04)—implementers and monitoring staff
- Target 2: Sociohealth authorities (regional health directorate [RHD], district executive team [DET], management committees, local elected officials)—key informants with relevant ministries’ representatives
- Target 3: community members (CSOs, health committees, health service users, health workers, etc)

We will make a purposive selection of the various targets likely to provide the best information about the AR project, implementation challenges, acceptability, use of devices, and application of results. Purposive sampling is used to select units that can provide the most insight into the issues that are important for the evaluation [54]. This type of sampling enables analytic generalizations (predictions of the probable portability of results grounded in a theoretical investigation of the effect of context and the factors that produce the direct effects). Criteria used for sampling will incorporate criteria for *inclusion* in a specific category as cited before, as opposed to cases that are *external* to a particular criterion [55]. In this way, we will ensure maximum variation within the targets (selection according to age, gender, participation or not in the AR, beneficiaries or not of KT training, etc). Particular attention will be paid to collecting the needs of specific populations, such as women, adolescents, and vulnerable persons.

The objective of theoretical saturation is to provide maximum detail about the AR project. It is thus a matter of selecting individuals who will ensure that all aspects of the AR project are included in the review to the point where no further substantive new knowledge is collected [56].

Private face-to-face interviews will be used to collect data. These will be conducted in French using the interview guide (Multimedia Appendix 3) developed based on the various conceptual frameworks cited before.

##### Data Analysis

We will use the procedure for analyzing qualitative data in health research developed by Gale et al [57]. Thus, all interviews will be recorded after obtaining the interviewees’ consent and then fully transcribed. The transcribed data will be coded using NVivo 12 software (QSR International) and then analyzed. Through content analysis, we will seek to understand general trends and particularities as well as recurrences and discrepancies by focusing on comparisons between stakeholder groups.

We will also undertake reflective analysis of the AR project to document successful practices and key implementation steps. This will take the form of workshops with the stakeholders to ask about their practices and the adaptations that enabled them to optimize the project implementation.

#### Economic Analysis (RQ5)

We will conduct a prospective cost analysis to assess the full economic costs associated with the AR project, collecting information as the project is being rolled out. Our aim will be to capture the full value of all resources used by any of the parties involved in the design and implementation of a given activity related to the AR. We will adopt a health system perspective, accounting for all costs incurred, whether for equipment, personnel mobilized, or activities.

##### Sampling and Collection Instruments

To trace all costs pertaining to the design and implementation of the AR project, we will adopt an activity-based costing (ABC) approach. Accordingly, we will start by mapping all microlevel activities related to the design and implementation of the initiative and then trace all resources consumed by these activities. We will supplement these first two steps by reviewing the complete documentation of the AR project and engaging in repeated exchanges with key stakeholders.

To attribute a value to either single resources (when possible) or complete activities (should the former not be possible), we will extract relevant cost information from the financial statements of the implementing partners. To estimate resource consumption for activities that cannot be traced in financial statements, we will conduct key informant interviews with implementation partners. We will obtain unit costs for valuation from existing costing sources in Burkina and, when necessary, complement that information with other information reported by key informants during interviews.

##### Data Analysis

To estimate the full economic cost of the AR project, we will multiply the resources consumed by the related unit costs and aggregate cost information across sources. In addition, to produce an accurate estimate of which activities and cost categories absorb the highest proportion of costs, we will disaggregate the analysis into both activity (eg, training, supervision) and cost categories (eg, mobile phone charges, personnel, transport). Our analysis will differentiate start-up costs from implementation costs.

#### KT Analysis (RQ6)

We will apply an integrated KT and knowledge translation strategy throughout the AR project. To measure the effects and processes of the KT approach to decision making, a mixed-methods approach (quantitative and qualitative) will also be used.

As a first step, to measure the overall satisfaction of participants in training and deliberative workshops, their appreciation of the content, and their suggestions for improvement [58] and to evaluate their intention to use the knowledge, we will undertake a *quantitative analysis*.

##### Study Population and Sampling

For the KT evaluation, the study population will comprise participants both in KT training sessions, including project team members (implementers and monitoring staff), and in deliberative workshops (sociohealth authorities—RHD, DET, management committees, local elected officials) and key informants from relevant ministries.

##### Collection Instruments

Data will be collected at the end of each training session and workshop and again 3 months later. Because of the difficulty of obtaining objective measures of change through observable behaviors, we will use indirect measures based on social cognition theories. The theory of planned behavior (TPB) measures the intention to use the information to change practices [59-61]. More specifically, the TPB states that the intention to engage in new behavior is predicted by the attitude toward the behavior to be adopted, the subjective social norm surrounding this behavior change, and the perceived control over the change to be made [62]. A reliable, innovative tool (α from 0.69 to 0.89 and G from 0.5 to 0.9) with 15 questions will be adapted to measure the participants’ intention to use the content presented [63]. It should be noted that this is essentially the same tool that will be used to evaluate training and deliberative workshops (see Multimedia Appendix 4).

##### Data Analysis

Data from the intent survey will be treated as continuous variables, and changes between different measurement times will be examined using a repeated-measures analysis of variance at the time variable. We will use the process tracing method [64] to explore information from the analysis of the quantitative questionnaires used to develop an initial portrait of potential users’ attitudes toward the research and their intention to use the items discussed in a deliberative workshop. This method will allow us to combine quantitative results with qualitative data from the interviews.

In addition to these data to be collected, it is essential to assess the use of knowledge for behavior change (activities carried out, decisions taken and implemented; RQ6); for this purpose, we will also perform a *qualitative analysis*.

##### Sampling Population and Collection Instruments

The KT assessment will also be based on qualitative interviews (see the interview guide in Multimedia Appendix 5). Three months after the activities, a semistructured interview grid will cover the important points for the evaluation, while leaving the necessary scope for further study of relevant elements that arise during the interviews [54]. The dimensions addressed in these interviews will allow us to complete and understand in greater depth the information collected using quantitative tools (reactions, attitudes, and intention to use). Results of the qualitative and quantitative analyses will be triangulated using a triangulation-convergence approach [45], in which the quantitative and qualitative components identify the same object in parallel, before being combined at the time of interpretation to increase the richness of the conclusions and contrast the information from complementary analytical angles.

In addition to formal interviews, we will also use qualitative data from informal interviews with different stakeholders, participant observations during the workshops, and activity reports (technical committee meetings, steering committee meetings, KT workshops, etc). These additional data will be used mainly to assess the impact of the KT strategy and data-sharing activities on the use of results for decision making by health and social authorities.

##### Qualitative Data Analysis

The qualitative material will be subjected to an iterative [56,65] thematic examination. The frequency of use and the nature of the knowledge used will be studied applying descriptive analyses, and analyses of variance will be used for comparative investigation of research use by demographic variables (user type, workplace, etc). Exploratory factor analysis will identify the internal structure of factors and their equivalence for respondents in various categories. The predictive power of these factors in knowledge use will then be examined by multiple regression.

#### Communication Campaigns to Publicize the TF Number’s Evaluation

##### Study Population and Sampling

For the campaign’s evaluation, the study population will be composed of sociohealth authorities (RHD, DET, management committees, local elected officials—critical informants with relevant ministries’ representatives) and community members (CSOs, health committees, health service users, health workers, etc).

##### Collection Instruments

Data will be collected at the end of each restitution session, which will be organized. The quantitative data will be derived from a questionnaire. The survey will measure the dimensions of interest, originality, visual appeal, instructive, and memorability of the campaigns.

##### Data Analysis

Quantitative data will be subject to automated descriptive analysis and disaggregated by sociodemographic characteristics (gender, groups, place of residence) and types of stakeholders involved (policymakers/CSO members/health center users). The variables of interest will be those asking for the stakeholders’ opinions on the effectiveness of the communication campaign to publicize the TF number. For all analyses, we plan to use R or computing and statistical graphs.

An even more in-depth evaluation of the effect of the communication campaigns on the use of the TF number (also through community work to raise awareness about health rights and duties) could be carried out by the project’s donor, World Affairs Canada, during its final evaluation.

### Patient and Public Involvement

This study protocol emerged during a series of meetings with authorities and stakeholders from the central government, the region, and areas in the targeted areas regarding their health and health care needs. This project is aimed at the population and health workers; they were indirectly involved in this study’s design. This project is based on evidence gathered through both a rigorous literature search and a previous pilot experiment in Burkina Faso that identified various subjects on which health center users and health professionals wanted to give their opinions on the health system. For the evaluation of the implementation, operation, use, acceptance, and efficiency of the digital device, the persons involved will mainly be the project team members, the sociohealth authorities, and community members. Finally, to maximize collaboration and to ensure that knowledge users are invested in using the study findings, a KT approach will be embedded throughout this project. Those who will benefit will be participants both in KT training sessions, including project team members, and in deliberative workshops and key informants from relevant ministries.

## Results

Data collection is in progress, the TF number was officially launched on July 1, 2020, and data collection is planned to continue throughout 2021. By using mixed methods, our AR will be approached from a variety of perspectives. Mixed methods will support us in combining the partial insights into sophisticated realities from qualitative inquiries with the data analyses produced by quantitative research.

### Data Dissemination

The data collected through the TF-IVS will be analyzed regularly, and the results will be disseminated on an ongoing basis to provide users of the health system and health authorities at all levels of the system with evidence to improve decision making. This sharing of results will be done through regular feedback to administrative personnel, health authorities, and CSOs. In addition to the activities planned in the KT strategy (as described in the Data Collection and Analysis section), the data-sharing strategy is defined as follows:

- An open data platform will be developed. It will be interoperable with the District Health Information System 2 (DHIS-2) of the National Health Information System (NHIS) to integrate the essential indicators. Presently, there are no secure servers in Burkina Faso capable of hosting 24 hours a day, 7 days a week, with optimal reliability the data that will be generated. Thus, initially, the data will be sent to France (OVH Roubaix), but a replication of the database will be set up in Burkina Faso. However, Burkina Faso’s health ministry and its health information systems services are currently building a data center to host all the country’s health data. Once the health ministry’s data center is accessible, the project’s data hosting will be migrated to the ministry’s system. An automated information-sharing module will reinforce this approach for members of the project’s technical and steering committees and other key stakeholders. The results will also be posted to the TOPICs project web portal, at which time they will be viewable by anyone in the population.
- A documentary film will be produced at the end of the project on the issues of governance and access to quality care for the most vulnerable. This film will be shared with the population, public decision makers, and other stakeholders.
- Reports on results, collection strategies, and means of expression will be prepared based on each of the technology assessments deployed. A capitalization report based on the evaluation reports will be prepared for the dissemination and popularization of the project results. This report will be made available to international, national, regional, and local institutions (policymakers, decision makers, CSOs, NGOs, associations, researchers, ICT/equality between women and men [EWM] experts).

### Ethical Considerations

Ethical approval was obtained from the Institutional Health Research Ethics Committee (IRSS) on September 24, 2019. The protocol research project registration number is 23–2019/CEIRES.

The use of the TF number will be free and voluntary. No personal information will be compiled from it. In this way, the data collected will be anonymous and kept confidential. The telephone operators who route the calls to the platform have contractually agreed to respect the confidentiality of the data and metadata of the calls. The software publisher has also contractually committed not to monitor or use identification data. Number capture, even if partial, was activated to identify the source telephone operators and quantify the share of each in terms of call flow (an important metric for the project). Finally, the software publisher has implemented additional security mechanisms for accessing and using the data with (1) secure individual access via a virtual private network (VPN), (2) a connection via a login and password to access the data, and (3) the implementation of a mechanism that allows, via pseudo-anonymization based on the SHA-256 hash algorithm, to transform each actual telephone number into a unique and irreversible digital fingerprint. A correspondence table has been set up in a different database, always with secure access.

All individual interviews will be conducted after the free and informed consent of the people interviewed. There is no risk associated with participating in this research project. The people who will be met will not receive any financial reward for their participation.

However, AR has policy and ethical implications that go well beyond the pure concerns of the free and informed consent of human subjects encountered in traditional research. Access to data; information use during and after investigation; the intellectual property of publishable materials resulting from the process; decision making throughout the study; and the impact of change on the individuals, organizations, and interest groups involved are essential issues that will inevitably confront the researchers.

## Discussion

### Importance of Principal Findings

mHealth technologies should contribute to improving the conditions of access to and provision of health care, while supplying a means to support public health policies [27]. Given human resource shortages and a lack of material and infrastructural support, the mobile device consequently would appear to reduce financial costs [66], be time effective, and overcome distances. Thus, mHealth constitutes a useful tool for reducing the technical and human burden of setting up surveys, making it possible to develop multiple data sources that are easily accessible and transferable between actors. Many such interventions [67] in sub-Saharan Africa offer information or prevention services [29] or give patients an opportunity to call a TF number and talk with a contact person. However, the use of SMS has shown its limitations [68], especially when addressing a rural population. In the literature, there are few interventions using ICTs associated with the IVS to improve health services access and health system governance at the same time.

### Strengths

Our protocol is innovative for several reasons: On the one hand, it proposes a technology associated with an evidence-based approach, adapted to the context, performed on a large scale, equitably and presumably at low cost; on the other hand, this approach is original in its AR dimension with communities and local NGOs, which includes a real KT component and spatial analysis of data, while using mixed methods of AR evaluation.

The IVS will be used to administer a survey to patients and health workers in the areas targeted by the project. This questionnaire was constructed through a rigorous scoping review that referenced the content offered to patients to measure satisfaction with the quality of and access to health care in West Africa [69-72]. It incorporates topics chosen by callers during the first phase of the TF-IVS pilot project [35] in connection with health system reforms, particularly in terms of the introduction of certain free services [73].

This AR project is to be carried out on a large territorial scale, integrating urban, rural, and periurban territories. The three zones combined cover 25% of the national population and are therefore intended to be reasonably representative of national opinion.

This questionnaire has been designed to be anonymous, adaptable, accessible, equitable, user friendly, and understandable (with clear instructions) to a heterogeneous population. For this purpose, a faithful translation into eight languages will make it possible to offer this service to the entire community of the targeted localities. This service will be available for all, everywhere and anytime, allowing people to give their opinion openly and anonymously.

The choice was made to conduct an audio survey rather than using SMS, because the former does not require any writing or reading skills and can thus be accessible to everyone. The necessary device is easy to use (the service is intuitive and easy to handle) and does not require a smartphone, just a touch phone.

Sustainability is an essential consideration when implementing an AR project [74]. Here, sustainability is supported on two fronts: (1) the call is free for the target populations and the user’s device is widely available (can be purchased or borrowed) and (2) costs are low, in terms of human and financial resources, for the organization that finances the project.

Moreover, touch-key technology enables additionally detailed, rigorous, and objective analysis, while being more efficient in terms of resources and time. It facilitates data management, in that data can be processed automatically online. Analyses will be executed over time and geographically. These spatiotemporal data represent a relevant added value for the evaluation of the project because they will measure spatial disparities in health care quality satisfaction. In addition, these types of mapping results are data that can be easily explained to decision makers and the public [75].

The uniqueness of this AR project lies, in particular, in the importance of making results available in several ways to different actors and in as many geographical areas as possible (at the regional and health district levels). This can be done by implementing an elaborate KT strategy based on the evidence regarding KT effectiveness [76]. The strategy will consist of (1) training [77] all implementing partners in techniques for making research results accessible to and usable by decision makers and the public from the start of the project and (2) presenting these results (problems identified by the AR and recommendations and proposals for action plans) at deliberative workshops, at the middle and end of the project, in the form of policy briefs [78] to complement the evidence produced by the project with other data from local expertise so that the resulting AR can be piloted in a feasible, applicable, and research-informed manner. The results of this work will be disseminated and shared through various information and awareness-raising strategies to encourage their adoption by health actors [79], and in particular by making data available on the platform to decision makers and other stakeholders in the health system.

However, for this KT strategy to be successful, it will be necessary to carry out in parallel the essential activity of making people aware of their health-related rights and duties, while informing them about the existence and the importance of using the TF number. Indeed, significant efforts will be made to create an enabling environment for people to understand and accept the system and be able to use it [80]. For that purpose, the AR project envisions involving local radio stations, communities, spiritual groups, trade unions, and local, religious, customary authorities.

The last main strength of this project is the evaluation approach, which is suited to complex problems. It will be subtle, in that it will be designed according to mixed methods, where each method will be applied rigorously in relation to the criteria set out before in the methodology. A convergent sort mode will be used to connect the data from a quantitative phase with the collection and analysis of data from another qualitative phase in order to be able to produce an assessment as relevant as possible throughout the AR project.

### Conclusions

To have a healthy society, everyone must be able to benefit from UHC, which implies an approach centered on the right to health. Achieving this goal requires not only strong political will but also the participation of all stakeholders. It is in this perspective that our AR project will take place. To improve health governance, we propose to exploit the technological potential of mobile telephony by offering a free service aimed at legitimately highlighting poor practices to serve as a basis to improve their management with the support of public opinion and health workers. In this way, the AR project would empower people to participate in democratic processes and to enjoy and exercise their human rights. Special attention will be paid to evaluating the project. By measuring its effectiveness, or lack thereof, we will be able to understand why, how, and under what conditions it can be most successful, based on the evidence produced. This approach could be relevant for supporting governments in their quest to improve population health.

## Appendix

Multimedia Appendix 1 Summary of the AR pilot project implemented in Burkina Faso in 2014 and 2015. AR: action research.

Multimedia Appendix 2 Interview guides.

Multimedia Appendix 3 TF-IVS questionnaire. TF-IVS: toll-free service coupled with an interactive voice server.

Multimedia Appendix 4 Interview guide for evaluation of the deliberative workshop.

Multimedia Appendix 5 Workshop evaluation questionnaire.

## Abbreviations

ABC: activity-based costing
AGIR-SD: Action-Gouvernance-Interaction-Renforcement, groupe de Santé en Développement
AR: action research
CFIR: Consolidated Framework for Implementation Research
CSO: civil society organization
DET: district executive team
DHIS2: District Health Information System 2
EWM: equality between women and men
GIS: geographic information system
HELP: Hilfe zur Selbsthilfe
ICT: information and communication technology
IEBHSR: Institutional Ethics Board for Health Sciences Research
IRD: Institut de recherche pour le développement
IRSS: Institutional Health Research Ethics Committee
JMS: Java Message Service
JSON: (IT) format for structured data exchange
KPI: key performance indicator
KT: knowledge transfer
LMIC: low- and middle-income country
mHealth: mobile health
NGO: nongovernmental organization
NHIS: National Health Information System
PHP: population health and policy
RAECP: Regulatory Authority for Electronic Communications and Posts
REST API: Representational State Transfer Application Programming Interface
RHD: regional health directorate
RQ: research question
SDG: Sustainable Development Goal
SMS: short message service
TF: toll free
TF-IVS: toll-free service coupled with an interactive voice server
TOPICs: TechnOlogie PartIcipation Citoyenne en santé
TIDieR: Template for Intervention Description and Replication
TPB: theory of planned behavior
UHC: universal health coverage
USI: International Health Unit
VPN: virtual private network
WHO: World Health Organization

## References

[1] DeSilva A, Valentine N, World Health Organization (2000). Global Programme on Evidence for Health Policy. (‎2000)‎. Measuring responsiveness: results of a key informants survey in 35 countries.

[2] Murray CJL, Evans DB, World Health Organization (2003). Global Programme on Evidence for Health Policy. (‎2003)‎. Health systems performance assessment : debates, methods and empiricism.

[3] Mirzoev T, Kane S (2017). What is health systems responsiveness? Review of existing knowledge and proposed conceptual framework. BMJ Glob Health.

[4] Siddiqi S, Masud TI, Nishtar S, Peters DH, Sabri B, Bile KM, Jama MA (2009). Framework for assessing governance of the health system in developing countries: gateway to good governance. Health Policy.

[5] Gilson L, Palmer N, Schneider H (2005). Trust and health worker performance: exploring a conceptual framework using South African evidence. Soc Sci Med.

[6] Van Belle S, Mayhew SH (2016). What can we learn on public accountability from non-health disciplines: a meta-narrative review. BMJ Open.

[7] Yuan B, Jian W, He L, Wang B, Balabanova D (2017). The role of health system governance in strengthening the rural health insurance system in China. Int J Equity Health.

[8] World Health Organization (2014). Health Systems Governance for Universal Health Coverage, Action Plan.

[9] Langseth P, Villadsen S, Lubanga F (1996). Civil service reform: a general view. Democratic Decentralization in Uganda: A New Approach to Local Governance.

[10] Sinha D (2008). Empowering communities to make pregnancy safer: an intervention in rural Andhra Pradesh. Health and Population Innovation Fellowship Programme Working Paper no. 5.

[11] Rifkin SB (1990). Community participation in maternal and child health/family planning programmes : an analysis based on case study materials. WHO IRIS.

[12] Standing H (2004). Understanding the 'demand side' in service delivery : definitions, frameworks and tools from the health sector.

[13] McGee R, Pettit J (2019). Power, Empowerment and Social Change.

[14] World Health Organization (2015). People-Centred and Integrated Health Services: An Overview of the Evidence.

[15] Boydell V, McMullen H, Cordero J, Steyn P, Kiare J (2019). Studying social accountability in the context of health system strengthening: innovations and considerations for future work. Health Res Policy Syst.

[16] Sebert Kuhlmann KA, Gullo S, Galavotti C, Grant C, Cavatore M, Posnock S (2017). Women's and health workers’ voices in open, inclusive communities and effective spaces (voices): measuring governance outcomes in reproductive and maternal health programmes. Dev Policy Rev.

[17] Mbwele B, Ide N, Mrema J, Ward Sarah A, Melnick J, Manongi R (2014). Learning from health care workers' opinions for improving quality of neonatal health care in kilimanjaro region, northeast Tanzania. Ann Med Health Sci Res.

[18] Janicijevic I, Seke K, Djokovic A, Filipovic T (2013). Healthcare workers satisfaction and patient satisfaction: where is the linkage?. Hippokratia.

[19] Nikic D, Arandjelovic M, Nikolic M, Stankovic A (2008). Job satisfaction in health care workers. Acta Medica Medianae.

[20] Bovier PA, Perneger TV (2003). Predictors of work satisfaction among physicians. Eur J Public Health.

[21] Lohmann J, Souares A, Tiendrebéogo J, Houlfort N, Robyn PJ, Somda SMA, De Allegri M (2017). Measuring health workers' motivation composition: validation of a scale based on Self-Determination Theory in Burkina Faso. Hum Resour Health.

[22] Fillol A, Lohmann J, Turcotte-Tremblay A, Somé P-A, Ridde V (2019). The importance of leadership and organizational capacity in shaping health workers' motivational reactions to performance-based financing: a multiple case study in Burkina Faso. Int J Health Policy Manag.

[23] Haas JS, Cook EF, Puopolo AL, Burstin HR, Cleary PD, Brennan TA (2000). Is the professional satisfaction of general internists associated with patient satisfaction?. J Gen Intern Med.

[24] Chang E, Cohen J, Koethe B, Smith K, Bir A (2017). Measuring job satisfaction among healthcare staff in the United States: a confirmatory factor analysis of the Satisfaction of Employees in Health Care (SEHC) survey. Int J Qual Health Care.

[25] Oyeyemi SO, Wynn R (2015). The use of cell phones and radio communication systems to reduce delays in getting help for pregnant women in low- and middle-income countries: a scoping review. Glob Health Action.

[26] Mericskay B, Roche S (2011). Cartographie 2.0: le grand public, producteur de contenus et de savoirs géographiques avec le web 2.0. Cybergeo: Eur J Geogr.

[27] Labrique AB, Vasudevan L, Kochi E, Fabricant R, Mehl G (2013). mHealth innovations as health system strengthening tools: 12 common applications and a visual framework. Glob Health Sci Pract.

[28] Hampshire K, Porter G, Mariwah S, Munthali A, Robson E, Owusu SA, Abane A, Milner J (2017). Who bears the cost of 'informal mhealth'? Health-workers' mobile phone practices and associated political-moral economies of care in Ghana and Malawi. Health Policy Plan.

[29] Duclos V, Yé M, Moubassira K, Sanou H, Sawadogo NH, Bibeau G, Sié Ali (2017). Situating mobile health: a qualitative study of mHealth expectations in the rural health district of Nouna, Burkina Faso. Health Res Policy Syst.

[30] Aranda-Jan CB, Mohutsiwa-Dibe N, Loukanova S (2014). Systematic review on what works, what does not work and why of implementation of mobile health (mHealth) projects in Africa. BMC Public Health.

[31] Hazra A, Khan ME, Mondal SK (2018). Mobile phone messaging to husbands to improve maternal and child health behavior in India. J Health Commun.

[32] Huang S, Li M (2017). Piloting a mHealth intervention to improve newborn care awareness among rural Cambodian mothers: a feasibility study. BMC Pregnancy Childbirth.

[33] Huq NL, Azmi AJ, Quaiyum MA, Hossain S (2014). Toll free mobile communication: overcoming barriers in maternal and neonatal emergencies in Rural Bangladesh. Reprod Health.

[34] Huda TM, Alam A, Tahsina T, Hasan MM, Khan J, Rahman MM, Siddique AB, Arifeen SE, Dibley MJ (2018). Mobile-based nutrition counseling and unconditional cash transfers for improving maternal and child nutrition in Bangladesh: pilot study. JMIR Mhealth Uhealth.

[35] Lechat L, Bonnet E, Queuille L, Traoré Z, Somé P-A, Ridde V (2019). Relevance of a toll-free call service using an interactive voice server to strengthen health system governance and responsiveness in Burkina Faso. Int J Health Policy Manag.

[36] D’Ostie-Racinea L, Dagenais C, Ridde V (2019). Examining conditions that influence evaluation use within a humanitarian non-governmental organization in Burkina Faso (West Africa). Syst Pract Action Res.

[37] Reason P, Bradbury H (2008). The Sage Handbook of Action Research: Participative Inquiry and Practice, 2nd ed.

[38] Craig P, DiRuggiero E, Frohlich K, Mykhalovskiy E, White M, on behalf of the Canadian Institutes of Health Research (CIHR)–National Institute for Health Research (NIHR) Context Guidance Authors Group (listed alphabetically): Campbell R, Cummins S, Edwards N, Hunt K, Kee F, Loppie C, Moore L, Ogilvie D, Petticrew M, Poland B, Ridde V, Shoveller J, Viehbeck S, Wight D (2018). Taking Account of Context in Population Health Intervention Research: Guidance for Producers, Users and Funders of Research.

[39] Campbell M, Katikireddi SV, Hoffmann T, Armstrong R, Waters E, Craig P (2018). TIDieR-PHP: a reporting guideline for population health and policy interventions. BMJ.

[40] Institut National de la Statistique et de la Démographie Résultats de l'Enquête Multisectorielle Continue (EMC) - Phase1.

[41] Autorité de Régulation des Communications Electroniques et des Postes (ARCEP) Market Observatory National Mobile Telephony Market Data 2nd Quarter 2019 Internet.

[42] National Institute of Statistics and Demography, Ministry of Economy and Finance (2009). Demographic Projections from 2007 to 2020 by Region and Province.

[43] Ministère de la santé (2019). Annuaire Statistique 2018. avec l’apui de l’UNICEF.

[44] National Statistics Council (2017). Annuaire Statistique 2017.

[45] Creswell J, Plano Clark VL (2011). Designing and Conducting Mixed Methods Research, 2nd ed.

[46] R Core Team (2018). R: A Language and Environment for Statistical Computing.

[47] Kulldorff M, Nagarwalla N (1995). Spatial disease clusters: detection and inference. Stat Med.

[48] Kulldorff M (2007). A spatial scan statistic. Commun Stat Theory Methods.

[49] Olivier de Sardan JP (2009). La rigueur du qualitatif. Les contraintes empiriques de l'interprétation socio-anthropologique.

[50] Stok FM, de Ridder DTD, de Vet E, Nureeva L, Luszczynska A, Wardle J, Gaspar T, de Wit JBF (2016). Hungry for an intervention? Adolescents' ratings of acceptability of eating-related intervention strategies. BMC Public Health.

[51] Diepeveen S, Ling T, Suhrcke M, Roland M, Marteau TM (2013). Public acceptability of government intervention to change health-related behaviours: a systematic review and narrative synthesis. BMC Public Health.

[52] Sekhon M, Cartwright M, Francis JJ (2017). Acceptability of healthcare interventions: an overview of reviews and development of a theoretical framework. BMC Health Serv Res.

[53] Damschroder LJ, Aron DC, Keith RE, Kirsh SR, Alexander JA, Lowery JC (2009). Fostering implementation of health services research findings into practice: a consolidated framework for advancing implementation science. Implement Sci.

[54] Patton MQ (1990). Qualitative evaluation and research methods. Designing Qualitative Studies, Qualitative Designs and Data Collection.

[55] Palinkas LA, Horwitz SM, Green CA, Wisdom JP, Duan N, Hoagwood K (2015). Purposeful sampling for qualitative data collection and analysis in mixed method implementation research. Adm Policy Ment Health.

[56] Miles M, Huberman AM (1994). Qualitative Data Analysis: An Expanded Sourcebook, 2nd ed.

[57] Gale NK, Heath G, Cameron E, Rashid S, Redwood S (2013). Using the framework method for the analysis of qualitative data in multi-disciplinary health research. BMC Med Res Methodol.

[58] Dagenais C, Dargis-Damphousse L, Dutil J (2011). The essential skills series in the essential skills series in evaluation: assessing the validity of the ES Participant Workshop Evaluation Questionnaire. Can J Program Eval.

[59] Armitage CJ, Conner M (2001). Efficacy of the theory of planned behaviour: a meta-analytic review. Br J Soc Psychol.

[60] Godin G, Kok G (1996). The theory of planned behavior: a review of its applications to health-related behaviors. Am J Health Promot.

[61] Foy R, Bamford C, Francis JJ, Johnston M, Lecouturier J, Eccles M, Steen N, Grimshaw J (2007). Which factors explain variation in intention to disclose a diagnosis of dementia? A theory-based survey of mental health professionals. Implement Sci.

[62] Ajzen I (1991). The theory of planned behavior. Organ Behav Hum Decis Process.

[63] Boyko JA, Lavis JN, Dobbins M, Souza NM (2011). Reliability of a tool for measuring theory of planned behaviour constructs for use in evaluating research use in policymaking. Health Res Policy Syst.

[64] Bennett A, Box-Steffensmeier JM, Brady HE, Collier D (2009). Process tracing: a Bayesian perspective. The Oxford Handbook of Political Methodology.

[65] Strauss A, Corbin J, Denzin NK, Lincoln YS (1994). Grounded theory methodology: an overview. Handbook of Qualitative Research.

[66] Larsen-Cooper E, Bancroft E, Rajagopal S, O'Toole M, Levin A (2016). Scale matters: a cost-outcome analysis of an m-Health intervention in Malawi. Telemed J E Health.

[67] Schuster C, Perez Brito C (2011). Cutting Costs, Boosting Quality and Collecting Data Real-Time: Lessons from a Cell Phone-Based Beneficiary Survey to Strengthen Guatemala's Conditional Cash Transfer Program.

[68] Chakraborty D, Gupta A, Seth A (2019). Experiences from a mobile-based behaviour change campaign on maternal and child nutrition in rural India.

[69] van Duong D, Binns CW, Lee AH, Hipgrave DB (2004). Measuring client-perceived quality of maternity services in rural Vietnam. Int J Qual Health Care.

[70] Haddad S, Fournier P, Potvin L (1998). Measuring lay people's perceptions of the quality of primary health care services in developing countries. Validation of a 20-item scale. Int J Qual Health Care.

[71] Ramanah R, Dumont A, Schepens F, Traore M, Gaye A, Schaal JP, Riethmuller D, Rude N (2014). Satisfaction with obstetrical care: development and validation of a scale on quality of care. Gynecol Obstet Fertil.

[72] Webster TR, Mantopoulos J, Jackson E, Cole-Lewis H, Kidane L, Kebede S, Abebe Y, Lawson R, Bradley EH (2011). A brief questionnaire for assessing patient healthcare experiences in low-income settings. Int J Qual Health Care.

[73] Ridde V, Yaméogo P (2018). How Burkina Faso used evidence in deciding to launch its policy of free healthcare for children under five and women in 2016. Palgrave Commun.

[74] Pluye P, Potvin L, Denis J, Pelletier J, Mannoni C (2005). Program sustainability begins with the first events. Eval Program Plann.

[75] Boyko JA, Lavis JN, Abelson J, Dobbins M, Carter N (2012). Deliberative dialogues as a mechanism for knowledge translation and exchange in health systems decision-making. Soc Sci Med.

[76] Dagenais C, Malo M, Ouimet M, Berthelette D, Ridde V, Robert (2013). Knowledge transfer on complex social interventions in public health: a scoping study. PLoS One.

[77] Tchameni Ngamo S, Souffez K, Lord C, Dagenais C (2016). Do knowledge translation (KT) plans help to structure KT practices?. Health Res Policy Syst.

[78] Manson H (2016). Systematic reviews are not enough: policymakers need a greater variety of synthesized evidence. J Clin Epidemiol.

[79] Lavis J, Permanand G, Oxman AD, Lewin S, Fretheim A (2009). SUPPORT tools for evidence-informed health policymaking (STP) 13: preparing and using policy briefs to support evidence-informed policymaking. Health Res Policy Sys.

[80] Shohet L, Renaud L (2006). Analyse critique des pratiques exemplaires sur la littératie en matière de santé. Can J Public Health.

