# Supplementary figures and images for: Using Information and Communication Technologies to Engage Citizens in Health System Governance in Burkina Faso: Protocol for Action Research
Source: JMIR Res Protoc. 2021 Nov 16;10(11):e28780. doi: 10.2196/28780 (PMC8663653; doi:10.2196/28780)

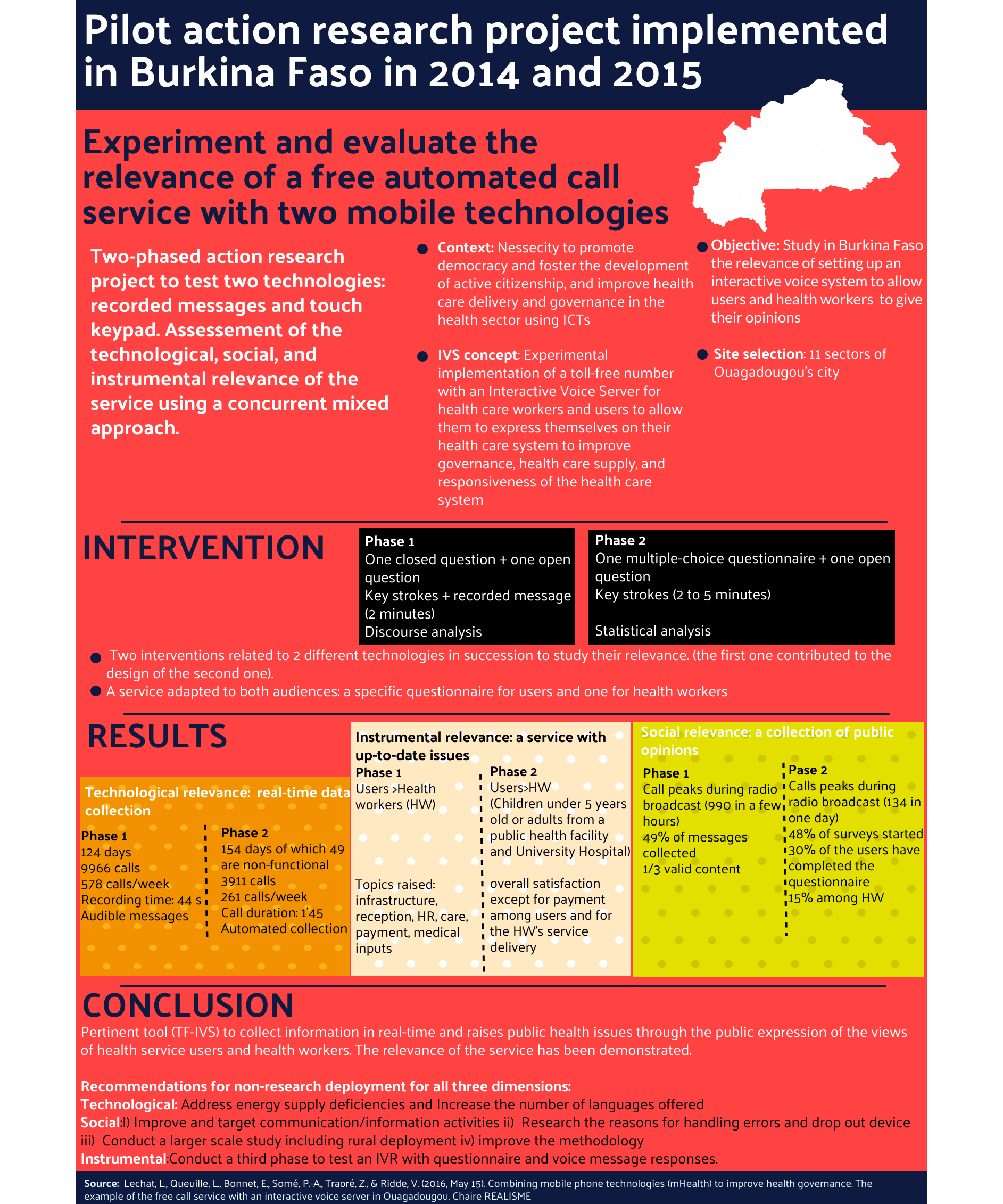

Supplement: Multimedia Appendix 1 [file resprot_v10i11e28780_app1.png]
