# Supplementary material for: Using Information and Communication Technologies to Engage Citizens in Health System Governance in Burkina Faso: Protocol for Action Research
Source: JMIR Res Protoc. 2021 Nov 16;10(11):e28780. doi: 10.2196/28780 (PMC8663653; doi:10.2196/28780)
Supplement: Multimedia Appendix 2 [file resprot_v10i11e28780_app2.docx]

**S2 Appendix_: Interview guides**

- **Interview guide: Project Team Members**

| **Date** |  |
| --- | --- |
| **Location** |  |
| **Name of respondent** |  |
| **Age** |  |
| **Start time** |  |
| **End time** |  |
| **Interviewer** |  |
| **Introduction (interviewer’s introduction)** |  |
| **Confidentiality and anonymity** | Any information you reveal to me will remain confidential. Under no circumstance will it be possible to identify you when the results of the study are presented or published. Only members of the Research Team will have access to the information for analysis.  Do you have any questions?  Please read and sign the consent form. |
| **Audio recording** | [Mention it and request permission.]  We can stop the recording at any moment for specific questions if you wish. You can also withdraw from the study at any time if you want to, without any consequences. |
| **Starting formula** | I will ask you some questions about the different aspects. What I am interested in are your reflections on the subject, so feel free to add anything you think is relevant. |
| **Themes** | **Questions of understanding** |
| **Knowledge of the actor’s function** | —Can you give me your profession/responsibility?  —Can you tell me about your experience on the job? |
| **Knowledge and acceptability of the TOPICs project** | —What do you think of this project? (Personal interest, interest in the system, interest in the community, etc.)  —Tell me what you know about it. (Actors, mechanisms, etc.)  —What is your involvement in its design and/or implementation? |
| **Use of the data produced** | —Tell us about the fate of the data collected —What is your opinion on the summaries made available?  —Talk about their usefulness for the health system and decision makers.  —What actions have been taken as a result of the different information in the districts?  —Tell us about the difficulties/facilities encountered in making the data available. (Platform, unsent mail, etc.)  —What solutions do you propose to correct this? |
| **Internal and external factors that may prevent access to and use of data** | —What barriers (other sources, lack of time, IT infrastructure problems, internet access, etc.) do you think may affect the use of data from the platforms for decision-making?  —In particular, has there been resistance from certain structures?  —What are the impacts of this resistance?  —What are the strategies to remedy it? |
| **Evaluation of the response of the health system** | —In your opinion, what changes has the project induced at the level of the SMCs, health agents, health center managers, districts, RHD, MOH?  —In your opinion, how did the project lead to these changes? |
| **Technological and organizational challenges of implementation** | —What are their strengths? Weaknesses?  —What are the technical difficulties in the installation, management, and maintenance of the system?  —What are the facilities/constraints for validation of project activities/results by the (technical committee, steering committee)?  —What are the possibilities for adaptation to better meet the needs of the health system? |
| **Effectiveness of ICTs compared to the use of old methods** | —What are the benefits/advantages of IVS in improving the health system?  —What are the drawbacks (technical, relational, institutional, etc.) in the implementation of this project?  —What is the contribution of these tools compared to the old methods (suggestion box….)?  —In what way can these tools replace the means of expression used by users? |
| **Conclusion** | We are at the end of my questions. Do you have any other concerns? |

- **Interview guide**: **Socio-sanitary authorities**

| **Date** |  |
| --- | --- |
| **Location** |  |
| **Region / District** |  |
| **Department / Municipality** |  |
| **Name of respondent** |  |
| **Age** |  |
| **Start time** |  |
| **End time** |  |
| **Interviewer** |  |
| **Introduction (interviewer’s introduction)** |  |
| **Confidentiality and anonymity** | Any information you reveal to me will remain confidential. Under no circumstance will it be possible to identify you when the results of the study are presented or published. Only members of the Research Team will have access to the information for analysis.  Do you have any questions?  Please read and sign the consent form. |
| **Audio recording** | [Mention it and request permission.]  We can stop the recording at any moment for specific questions if you wish. You can also withdraw from the study at any time if you want to, without any consequences. |
| **Starting formula** | I will ask you some questions about the different aspects. What I am interested in are your reflections on the subject, so feel free to add anything you think is relevant. |
| **Themes** | **Questions of understanding** |
| **Knowledge of the actor’s function** | —Can you give me your profession/responsibility?  —Can you tell me about your experience in that position? |
| **History of citizen participation** | — Before the implementation of the TF-IVS, how user could give their opinion on the health care system to contribute to its improvement;?  —What were the difficulties encountered?  —How were users' needs/opinions integrated into the health offers?  —What were the limits of your collaboration with users/health workers? |
| **Knowledge and acceptability of the TOPICs project** | —How did you find out about the TOPICs project?  —Tell me what you know about it. (Actors, mechanisms, etc.)  —What do you think of this project? (Personal interest, interest in the system, interest in the community, etc.).  —What does a person like you gain/lose by getting involved in the implementation of this project?  —What is your involvement in its design and/or implementation? |
| **Use of data produced** | —Tell us about the fate of the collected data.  —Can you use the summaries of the compiled data  —If so, how?  —What is your opinion of the overviews provided?  —Tell us about their utility for you/your organization.  —Do you find them satisfactory for your needs?  —What actions have been taken as a result of the different information?  —Have you ever used project data to guide a decision/action?  —Tell us about the difficulties/ease encountered in accessing the data? (Platform unreachable, mail not sent, etc.).  —What solutions would you propose to correct this? |
| **Factors favoring the use of results** | What factors motivate you to make decisions based on the results?  Explain:   - Accountability - Pressure from the health authorities - Pressure from civil society organizations - Concern to improve indicators - Data reliability |
| **Internal and external factors that may prevent access to and use of data in your unit** | —In your opinion, what are the barriers (other sources, lack of time, computer infrastructure problems, internet access, etc.) that may affect the use of data from the platforms for decision-making?  —Specifically, has there been resistance from certain structures under trusteeship.?  —What are the impacts of this resistance?  —What are the strategies to remedy it? |
| **Evaluation of the health system’s response** | —In your opinion, what changes has the project induced at the level of health workers, health center managers, SMCs, district, MOH?  —In your opinion, how did the project lead to these changes? |
| **Added value of the project's ICT innovation** | —What do you think of the ICT collection tools used by the project?  —What is the impact of the different technological tools used on the users/providers relationship?  —What is the impact of ICT on the response?  —What is the impact on the integration of users' needs? |
| **Effectiveness of ICTs compared to the use of old methods** | —What are the benefits/advantages of TF-IVS for improving the health system?  —What are the contributions of these tools compared to the old expression methods (suggestion box…)?  —How can these tools replace the means of expression used by the users? |
| **Conclusion** | We are at the end of my questions. Do you have any other concerns? |

- **Interview guide**: **Community organizations in civil society, health committees, health services users, health workers, etc.)**

| **Date** |  |
| --- | --- |
| **Location** |  |
| **Region / District** |  |
| **Department / Municipality** |  |
| **Name of respondent** |  |
| **Age** |  |
| **Start time** |  |
| **End time** |  |
| **Interviewer** |  |
| **Introduction (interviewer’s introduction)** |  |
| **Confidentiality and anonymity** | Any information you reveal to me will remain confidential. Under no circumstance will it be possible to identify you when the results of the study are presented or published. Only members of the Research Team will have access to the information for analysis.  Do you have any questions?  Please read and sign the consent form. |
| **Audio recording** | [Mention it and request permission.]  We can stop the recording at any moment for specific questions if you wish. You can also withdraw from the study at any time if you want to, without any consequences. |
| **Starting formula** | I will ask you some questions about the different aspects. What I am interested in are your reflections on the subject, so feel free to add anything you think is relevant. |
| **Themes** | **Questions of understanding** |
| **Knowledge of the actor’s function** | —Can you give me your profession/responsibility?  —Can you tell me about your experience in that position? |
| **History of citizen participation** | —What was Before the implementation of the TF-IVS, how user could give their opinion on the health care system to contribute to its improvement?  —What were the difficulties encountered?  —How were users' needs/opinions integrated into the health offers?  —What were the limits of your collaboration with users/health workers? |
| **Knowledge and acceptability of the TOPICs project** | —How did you find out about the TOPICs project?  —Tell me what you know about it. (Actors, mechanisms, etc.)  —What do you think of this project? (Personal interest, interest in the system, interest in the community, etc.).  —What does a person like you gain/lose by getting involved in the implementation of this project?  —What is your involvement in its design and/or implementation? |
| **Use of the data produced** | —Can you use the summaries of the compiled data?  —If so, how?  —What is your opinion on the overviews provided?  —Tell us about their utility for you/your organization.  —Do you find them satisfactory for your needs?  —What actions have been taken as a result of the different information?  —Have you ever used project data to guide a decision/action?  —Tell us about the difficulties/ease encountered in accessing the data. (Platform unreachable, mail not sent, etc.).  —What solutions would you propose to correct this? |
| **Internal and external factors that may prevent access to and use of data in your unit** | —In your opinion, what are the barriers (other sources, lack of time, computer infrastructure problems, internet access ...) that may affect the use of TF-IVS?  —Specifically, has there been resistance from certain structures?  —What are the impacts of this resistance?  —What are the strategies to remedy it? |
| **Evaluation of the health system’s response** | —In your opinion, what changes has the project induced at the level of health workers, health center managers, SMCs, district, MOH?  —In your opinion, how did the project lead to these changes? |
| **Added value of the project's ICT innovation** | —What do you think of the ICT collection tools used by the project?  —What is the impact of the different technological tools used on the users/providers relationship?  —What is the impact of ICT on the response?  —What is the impact on the integration of users' needs? |
| **Effectiveness of ICTs compared to the use of old methods** | —What are the benefits/advantages of TF-IVS in improving the health system?  —What are the contributions of these tools compared to the old expression methods (suggestion box…)?  —How can these tools replace the means of expression used by the users?  —What can be done to improve the efficiency of the TF-IVS? |
| **Conclusion** | We are at the end of my questions. Do you have any other concerns? |
