# Supplementary material for: Using Information and Communication Technologies to Engage Citizens in Health System Governance in Burkina Faso: Protocol for Action Research
Source: JMIR Res Protoc. 2021 Nov 16;10(11):e28780. doi: 10.2196/28780 (PMC8663653; doi:10.2196/28780)
Supplement: Multimedia Appendix 3 [file resprot_v10i11e28780_app3.docx]

**S3 Appendix: TF-IVS Questionnaire**

|  | **USERS Questionnaire** |  | **HEALTH PROFESSIONALS Questionnaire** |
| --- | --- | --- | --- |
| U.1 | Hello.  This is a computer talking to you. Choose your language: | HP.1 | = U.1 |
| U.2 | 1) French, press 1  2) Dioula, press 2  3) Mossi, press 3  4) Fulfulde, press 4  5) Lobiri, press 5  6) Djan, press 6  7) Mande, press 7  8) Senufo, press 8  9) Bwamu, press 9 | HP.2 | = U.2 |
| U.3 | The computer didn’t recognize your answer. Choose your language: | HP.3 | = U.3 |
| U.4 | = U.2 | HP.4 | = U.2 |
| U.5 | Without a valid answer to this question, you can’t access the survey. Call another time if you wish. We wish you good health | HP.5 | = U.5 |
| U.6 | You are calling to give your opinion on the health care system anonymously. If you are: | HP.6 | = U.6 |
| U.7 | 1) A health services user, press 1  2) A health care professional, press 2 | HP.7 | = U.7 |
| U.8 | The computer didn’t recognize your answer. If you are: | HP.8 | = U.8 |
| U.9 | = U.7 | HP.9 | = U.7 |
| U.10 | = U.5 | HP.10 | = U.5 |
| U.11 | In the past 12 months, how many times have you gone to a health facility to receive health care for yourself? | HP.11 | You are a health professional. You want to share your opinion on the Burkinabe health system |
| U.12 | 1) Never, press 1  2) 1 to 2 times, press 2  3) 3 or more times, press 3 | HP.12 | In which health sector do you work? |
| U.13 | The computer didn’t recognize your answer. In the past 12 months, how many times have you gone to a health facility to receive health care for yourself? | HP.13 | 1) Public, press 1  2) Private for-profit, press 2  3) Private non-profit, press 3  4) Traditional, press 4  5) Government partner, press 5  6) Other, press 6 |
| U.14 | = U.12 | HP.14 | The computer didn’t recognize your answer. In which health sector do you work? |
| U.15 | You have used health services, and you want to tell us what you think. | HP.15 | = HP.13 |
| U.16 | Your opinion concerns a health facility that is: | HP.16 | You work in health care as: |
| U.17 | 1) Public, press 1  2) Private, press 2  3) You don't know, press 3 | HP.17 | 1) A health care worker, press 1  2) A manager, technical support staff, or program manager, press 2  3) An administrator, press 3  4) Other, press 4 |
| U.18 | The computer didn’t recognize your answer. Your opinion concerns a health facility that is: | HP.18 | The computer didn’t recognize your answer. You work in health care as: |
| U.19 | = U.17 | HP.19 | = HP.17 |
| U.20 | Your opinion concerns a health facility of the type: | HP.20 | At what level do you practice? |
| U.21 | 1) Hospital or polyclinic, press 1  2) MCA/MC or clinic, press 2  3) HSPC, dispensary or nursing office, press 3  4) Other, press 4 | HP.21 | 1) HSPC, press 1  2) MCA/MC or clinic, press 2  3) Hospital or polyclinic, press 3  4) National, press 4  5) International, press 5 |
| U.22 | The computer didn’t recognize your answer. Your opinion concerns a health facility of the type: | HP.22 | The computer didn’t recognize your answer. At what level do you practice? |
| U.23 | = U.21 | HP.23 | = HP.21 |
| U.24 | The health facility you want to talk about is located in: | HP.24 | The questionnaire is constructed in line with the ESSENTIAL ELEMENTS of a HEALTH SYSTEM. You will only be able to provide a maximum of two opinions. Choose what you think is most important. |
| U.25 | 1) Ouagadougou, press 1  2) Diébougou, press 2  3) Bobo Dioulasso, press 3  4) Houndé, press 4  5) Orodara, press 5  6) Others, press 6 | HP.25 | If you wish to give your first opinion about: |
| U.26 | The computer didn’t recognize your answer. The health facility you want to talk about is located in: | HP.26 | 1) Leadership and governance, press 1  2) Services, press 2  3) Free care, press 3  4) Human resources, press 4  5) Drugs and medical products, press 5  6) Infrastructure, equipment and maintenance, press 6  7) The health information system, press 7  8) Research, press 8  9) Financing, press 9 |
| U.27 | = U.25 | HP.27 | The computer didn’t recognize your answer. If you want to give your first opinion about: |
| U.28 | If you live in Ouagadougou: | HP.28 | = HP.26 |
| U.29 | 1) Press the number of your sector of residence  2) If you are in an undivided area, press 0 | HP.29 | = U.45 |
| U.30 | The computer didn’t recognize your answer. If you live in Ouagadougou: | HP.30 | For <PREVIOUS ANSWER CHOSEN (Cf. HP.30.1 to HP.30.9 beginning at line 160)>, your opinion concerns: |
| U.31 | = U.29 |  | If LEADERSHIP AND GOVERNANCE |
| U.32 | If you live in Bobo Dioulasso: | HP.31 | 1) Regulations, press 1  2) Health policies, press 2  3) Ministry of Health leadership, press 3  4) Help from financial partners, press 4  5) Accountability, press 5  6) Health system responsiveness, press 6  7) Coordination of technical and financial partners, press 7  8) The closing of unauthorized private practices, press 8 |
| U.33 | = U.29 |  | If SERVICES |
| U.34 | The computer didn’t recognize your answer. If you live in Bobo Dioulasso: | HP.32 | 1) Their availability, press 1  2) Their accessibility, press 2  3) Their organization, press 3  4) Their quality, press 4  5) Their effectiveness, press 5  6) Other, press 6 |
| U.35 | = U.29 |  | If FREE CARE |
| U.36 | If you used health services for: | HP.33 | 1) For pregnant women, press 1  2) For children under five years old, press 2  3) For indigents, press 3 |
| U.37 | 1) A pregnant woman, press 1  2) A child under five years old, press 2  3) A child over five years old, press 3  4) Yourself or an adult, press 4 | HP.34 | The computer didn’t recognize your answer. Your opinion concerns: |
| U.38 | The computer didn’t recognize your answer. If you used health services for: | HP.35 | HP.33 |
| U.39 | = U.37 | HP.36 | In your opinion, does free health care really exist? |
| U.40 | The questionnaire is constructed according to the PATIENT’S VISIT in a health facility. You will only be able to give a maximum of two opinions. Choose what you think is most important. | HP.37 | 1) Yes, press 1  2) No, press 2 |
| U.41 | If you wish to give your first opinion about: | HP.38 | If according to you, free health care isn’t effective or available, is it for reasons of: |
| U.42 | 1) Reception, press 1  2) Consultation, press 2  3) Drugs and pharmaceuticals, press 3  4) Price, press 4  5) Free treatment, press 5 | HP.39 | 1) Out of stock, type 1  2) Delay in the reimbursement of costs incurred by the care, from the government to the health center,type 2  3) Work overload, type 3  4) Other reasons, type 4  5) You don’t know, type 5 |
| U.43 | The computer didn’t recognize your answer. If you wish to give your first opinion about: | HP.40 | The computer didn’t recognize your answer, it’s for reasons of: |
| U.44 | = U.42 | HP.41 | =HP.39 |
|  |  |  | If HUMAN RESOURCES |
| U.45 | For <PREVIOUS ANSWER CHOSEN (Cf. U46.1 to U.46.5 beginning at line 160)>, your opinion concerns: | HP.42 | 1) Their training, press 1  2) Their quantity, press 2  3) Their diversity, press 3  4) Their distribution, press 4  5) Their management, press 5  6) Their remuneration, press 6  7) Their performance, press 7  8) Their career, press 8  9) Other, press 9 |
|  | If RECEPTION |  | If DRUGS AND MEDICAL PRODUCTS |
| U.46 | 1) The building, press 1  2) The staff, press 2  3) The waiting time, press 3  4) The organization of services, press 4  5) Other, press 5 | HP.43 | 1) Their national production, press 1  2) Their supply, press 2  3) Their quality, press 3  4) Their availability, press 4  5) Their storage, press 5  6) Their use, press 6  7) Their affordability, press 7  8) Other, press 8 |
|  | If CONSULTATION |  | If INFRASTRUCTURE, EQUIPMENT & MAINTENANCE |
| U.47 | 1) Your physical exam, press 1  2) Explanations given about the illness, press 2  3) Explanations given on medications, tests, etc., press 3  4) The duration of the consultation, press 4  5) The respect you were accorded, press 5 6) The equipment, press 6  7) Other, press 7 | HP.44 | 1) Infrastructure, press 1  2) Equipment, press 2  3) Maintenance, press 3  4) All three, press 4 |
|  | If DRUGS AND PHARMACEUTICALS |  | If HEALTH INFORMATION SYSTEM |
| U.48 | 1) Their availability at the health facility, press 1  2) The explanations given by the depot manager, press 2  3) The packaging of the drugs, press 3  4) Other, press 4 | HP.45 | 1) Data quality, press 1  2) Validity of the results produced, press 2  3) Results relevance, press 3  4) Results use, press 4  5) Other, press 5 |
|  | If PRICE |  | If RESEARCH |
| U.49 | 1) The price of the consultation, press 1  2) The price of drugs, press 2  3) The price of tests and/or other examinations, press 3  4) Other, press 4 | HP.46 | 1) Relevance of the research for the country, press 1  2) Validity of research results, press 2  3) Accessibility of research results, press 3  4) Diffusion to decision-makers, press 4  5) Use of results by decision-makers, type 5  6) Research financing, press 6 |
|  | If FREE CARE |  | If FINANCING |
| U.50 | 1) Free care for pregnant women, press 1  2) Free care for children under 5 years old, press 2  3) Free care for indigents, press 3 | HP.47 | 1) Health facility budgets, press 1  2) Fee-for-service patient care, press 2  3) Free care for indigents, press 3  4) Grant programs, press 4  5) Mutual health insurance, press 5  6) Other, press 6 |
| U.51 | In your opinion, does free care really exist? | HP.48 | = U.45 |
| U.52 | 1) Yes, press 1  2) No, press 2 | HP.49 | = HP.31 |
| U.53 | The computer didn’t recognize your answer. Your opinion is: | HP.50 | = HP.32 |
| U.54 | = U.53 | HP.51 | = HP.33 |
| U.55 | The computer didn’t recognize your answer. Your opinion is: | HP.52 | = HP.34 |
| U.56 | = U.47 | HP.53 | = HP.33 |
| U.57 | = U.48 | HP.54 | = HP.36 |
| U.58 | = U.49 | HP.55 | = HP.37 |
| U.59 | = U.50 | HP.56 | = HP.38 |
| U.60 | = U.51 | HP.57 | = HP.39 |
| U.61 | = U.52 | HP.58 | = HP.40 |
| U.62 | = U.53 | HP.59 | = HP.39 |
| U.63 | = U.54 | HP.60 | = HP.42 |
| U.64 | = U.53 | HP.61 | = HP.43 |
| U.65 | = U.45 | HP.62 | = HP.44 |
| U.66 | For <PREVIOUS ANSWER CHOSEN (Cf. U.67.1 to U.67.5 beginning at line 166)>, if your opinion is: | HP.63 | = HP.45 |
| U.67 | 1) Very positive, press 1  2) Positive, press 2  3) Negative, press 3  4) Very negative, press 4 | HP.64 | = HP.46 |
| U.68 | The computer didn’t recognize your answer. If your opinion is: | HP.65 | = HP.47 |
| U.69 | = U.68 | HP.66 | = U.45 |
| U.70 | = U.45 | HP.67 | For <PREVIOUS ANSWER CHOSEN (Cf. HP.67.1 to HP.67.8 beginning at line 170)>, your opinion concerns: |
| U.71 | If the caller chose <Price> and answered with <NEGATIVE> or <VERY NEGATIVE> input | HP.68 | 1) High strength, press 1  2) Strength, press 2  3) Weakness, press 3  4) Great weakness, press 4 |
| U.72 | You have expressed a <NEGATIVE/ VERY NEGATIVE> opinion regarding the <Price>. Do you think you paid more than you should have? | HP.69 | The computer didn’t recognize your answer. Your opinion concerns: |
| U.73 | 1) Yes, because too expensive, press 1  2) Yes, because of corruption, press 2  3) No, press 3 | HP.70 | = HP.68 |
| U.74 | The computer didn’t recognize your answer. You expressed a <NEGATIVE/ VERY NEGATIVE > opinion concerning the <Price>. Do you think you paid more than you should have? | HP.71 | = U.45 |
| U.75 | = U.74 | HP.72 | Do you wish to give a second opinion on another essential element of the health care system? |
| U.76 | Did any of the amounts you had to pay force you to forego all or part of the care, medication, and/or other services? | HP.73 | = HP.37 |
| U.77 | = U.53 | HP.74 | The computer didn’t recognize your answer. Do you wish to give a second opinion on another essential element of the health system? |
| U.78 | The computer didn’t recognize your answer. Did any of the amounts you had to pay force you to forego all or part of the care, medication, and/or other services? | HP.75 | = HP.37 |
| U.79 | = U.53 | HP.76 | If YES |
| U.80 | Do you wish to give a second opinion on another part of the service received? | HP.77 | On which essential element of the health care system do you wish to give your second opinion? |
| U.81 | = U.53 | HP.78 | = HP.26 |
| U.82 | The computer didn’t recognize your answer. Do you wish to give a second opinion on another part of the service received? | HP.79 | The computer didn’t recognize your answer. On which essential element of the health care system do you wish to give your second opinion? |
| U.83 | = U.53 | HP.80 | = HP.26 |
| U.84 | If YES : | HP.81 | = HP.30 |
| U.85 | What aspect of your visit would you like to give your second opinion about? | HP.82 | = HP.31 |
| U.86 | = U.42 | HP.83 | = HP.32 |
| U.87 | The computer didn’t recognize your answer. What aspect of your visit do you want to give your second opinion about? | HP.84 | = HP.33 |
| U.88 | = U.42 | HP.85 | = HP.34 |
| U.89 | = U.46 | HP.86 | = HP.33 |
| U.90 | = U.47 | HP.87 | = HP.36 |
| U.91 | = U.48 | HP.88 | = HP.37 |
| U.92 | = U.49 | HP.89 | = HP.38 |
| U.93 | = U.50 | HP.90 | = HP.39 |
| U.94 | = U.51 | HP.91 | = HP.40 |
| U.95 | = U.52 | HP.92 | = HP.39 |
| U.96 | = U.53 | HP.93 | = HP.42 |
| U.97 | = U.54 | HP.94 | = HP.43 |
| U.98 | = U.53 | HP.95 | = HP.44 |
| U.99 | = U.45 | HP.96 | = HP.45 |
| U.100 | = U.47 | HP.97 | = HP.46 |
| U.101 | = U.48 | HP.98 | = HP.47 |
| U.102 | = U.49 | HP.99 | = U.45 |
| U.103 | = U.50 | HP.100 | = HP.31 |
| U.104 | = U.51 | HP.101 | = HP.32 |
| U.105 | = U.52 | HP.102 | = HP.33 |
| U.106 | = U.53 | HP.103 | = HP.34 |
| U.107 | = U.54 | HP.104 | = HP.33 |
| U.108 | = U.53 | HP.105 | = HP.36 |
| U.109 | = U.45 | HP.106 | = HP.37 |
| U.110 | = U.67 | HP.107 | = HP.38 |
| U.111 | = U.68 | HP.108 | = HP.39 |
| U.112 | = U.69 | HP.109 | = HP.40 |
| U.113 | = U.68 | HP.110 | = HP.39 |
| U.114 | = U.45 | HP.111 | = HP.42 |
| U.115 | = U.72 | HP.112 | = HP.43 |
| U.116 | = U.73 | HP.113 | = HP.44 |
| U.117 | = U.74 | HP.114 | = HP.45 |
| U.118 | = U.75 | HP.115 | = HP.46 |
| U.119 | = U.74 | HP.116 | = HP.47 |
| U.120 | = U.77 | HP.117 | For <PREVIOUS RESPONSE CHOSEN (Cf. HP.67.1 to HP.67.8 beginning at line 170)>, your opinion concerns: |
| U.121 | = U.53 | HP.118 | HP.68 |
| U.122 | On your last visit to a health care facility, how well did the health care staff take the time to listen to you? | HP.119 | The computer didn’t recognize your answer. Your opinion concerns: |
| U.123 | 1) Very good listening, press 1  2) Good listening, press 2  3) Bad listening, press 3  4) Very bad listening, press 4 | HP.120 | = HP.68 |
| U.124 | The computer didn’t recognize your answer. On your last visit to a health care facility, how well did the health care staff take the time to listen to you? | HP.121 | = U.45 |
| U.125 | = U.124 | HP.122 | In which health region do you practice? |
| U.126 | How confident are you that if you get very sick tomorrow, you will be able to receive adequate treatment from the health care system? | HP.123 | 1) Central health region, press 1  2) Haut Bassins health region, press 2  3) Southwest health region, press 3  4) Other health region, press 4 |
| U.127 | 1) Very confident, press 1  2) Confident, press 2  3) Not confident, press 3  4) Not at all confident, press 4 | HP.124 | If you are in the Central health region, what health district do you work in? |
| U.128 | The computer didn’t recognize your answer. How confident are you that if you get very sick tomorrow, you will be able to receive adequate treatment from the health care system? | HP.125 | 1) Bogodogo health district, press 1  2) Boumioulgou health district, press 2  3) Signoghin health district, press 3  4) Nongremasson health district, press 4  5) Baskuy health district, press 5  6) Other health district, press 6 |
| U.129 | = U.128 | HP.126 | If you are in the Haut Bassins health region, what health district do you work in? |
| U.130 | Please indicate: | HP.127 | 1) Dafra health district, press 1  2) Do health district, press 2  3) Dandé health district, press 3  4) Léna health district, press 4  5) Karangasso-vigué health district, press 5  6) Houndé health district, press 6  7) N'dorola health district, press 7  8) Orodara health district, press 8  9) Other health district, press 9 |
| U.131 | 1) If you are a woman, press 1  2) If you are a man, press 2  3) If you don’t want to answer, press 3 | HP.128 | If you are in the Southwest health region, what health district do you work in? |
| U.132 | The computer didn’t recognize your answer. Please indicate: | HP.129 | 1) Diébougou health district, press 1  2) Other health district, press 2 |
| U.133 | = U.132 | HP.130 | The computer didn’t recognize your answer. In which health region do you practice? |
| U.134 | Please indicate if your age is: | HP.131 | = HP.123 |
| U.135 | 1) Less than 15 years old, press 1  2) Between 15 and 19 years old, press 2  3) Between 20 years and 40 years, press 3  4) If you are over 40 years old, press 4  5) If you do not wish to answer, press 5 | HP.132 | = HP.124 |
| U.136 | The computer didn’t recognize your answer. Please indicate if your age is: | HP.133 | = HP.125 |
| U.137 | = U.136 | HP.134 | = HP.126 |
| U.138 | We are at the end of the survey. Would you like to receive information on health rights and duties? | HP.135 | = HP.127 |
| U.139 | = U.53 | HP.136 | = HP.128 |
| U.140 | The computer didn’t recognize your answer. Would you like to listen to a recorded message on your health rights and duties? | HP.137 | = HP.129 |
| U.141 | = U.53 | HP.138 | How many years have you been working in the health care sector? |
| U.142 | If yes, please specify: | HP.139 | 1) Less than 5 years, press 1  2) Between 5 and 10 years, press 2  3) More than 10 years, press 3 |
| U.143 | Information messages on health rights and duties | HP.140 | The computer didn’t recognize your answer. How many years have you been working in the health care sector? |
| U.144 | Thank you, and congratulations on your contribution. You can consult the web page www.agir-sd.org to obtain all the information concerning your health rights and duties. The results produced from your answers will be shared and discussed within a few months with all the actors of the health system, including users, of course. | HP.141 | = HP.138 |
|  |  | HP.142 | We’re at the end of the survey. Would you like to receive information on your health rights and duties? |
|  |  | HP.143 | = HP.37 |
|  |  | HP.144 | The computer didn’t recognize your answer. Would you like to listen to a recorded message on your health rights and duties? |
|  |  | HP.145 | = HP.37 |
|  |  | HP.146 | If YES |
|  |  | HP.147 | Information messages on health rights and duties |
|  |  | HP.148 | Thank you, and congratulations on your contribution. You can consult the web page www.agir-sd.org to obtain all the information concerning your health rights and duties. The results produced from your answers will be shared and discussed within a few months with all the actors of the health system, including users, of course. |
|  |  |  |  |
| **Development of possible audio messages for U.46** | | **Development of possible audio messages for HP.30** | |
| U.46.1 | For <Reception>, your opinion concerns: | HP.30.1 | For <Leadership and governance>, your opinion concerns: |
| U.46.2 | For <Consultation>, your opinion concerns: | HP.30.2 | For <Health care>, your opinion concerns: |
| U.46.3 | For <Drugs and Pharmaceuticals>, your opinion concerns: | HP.30.3 | For <Free care>, your opinion concerns: |
| U.46.4 | For <Price>, your opinion concerns: | HP.30.4 | For <Human resources>, your opinion concerns: |
| U.46.5 | For <Free care>, your opinion concerns: | HP.30.5 | For <Drugs and medical products>, your opinion concerns: |
| **Development of possible audio messages for U.46** | | HP.30.6 | For <Infrastructure, equipment and maintenance>, your opinion concerns: |
|  | If RECEPTION | HP.30.7 | For <Health information system>, your opinion concerns: |
| U.67.1.1 | For <The building>, your opinion concerns: | HP.30.8 | For <Research>, your opinion concerns: |
| U.67.1.2 | For <The equipment>, your opinion concerns: | HP.30.9 | For <Financing>, your opinion concerns: |
| U.67.1.3 | For <The staff>, your opinion concerns: | **Development of possible audio messages for HP.67** | |
| U.67.1.4 | For <The waiting time>, your opinion concerns: |  | If LEADERSHIP and GOVERNANCE |
| U.67.1.5 | For <The organization of services>, your opinion concerns: | HP.67.1.1 | For <Regulation>, your opinion concerns: |
| U.67.1.6 | For <Other>, your opinion concerns: | HP.67.1.2 | For <Health policy>, your opinion concerns: |
|  | If CONSULTATION | HP.67.1.3 | For <Ministry of Health leadership>, your opinion concerns: |
| U.67.2.1 | For <Your physical exam>, your opinion concerns: | HP.67.1.4 | For <Funding partner support>, your opinion concerns: |
| U.67.2.2 | For <The explanations about the illness>, your opinion concerns: | HP.67.1.5 | For <Accountability>, your opinion concerns: |
| U.67.2.3 | For <The explanations on the medications, tests, etc.,.>, your opinion concerns: | HP.67.1.6 | For <Health system responsiveness>, your opinion concerns: |
| U.67.2.4 | For <The length of the consultation>, your opinion concerns: | HP.67.1.7 | For <Coordination of technical and financial partners>, your opinion concerns: |
| U.67.2.5 | For <The respect you were accorded>, your opinion concerns: | HP.67.1.8 | For <The closing of unauthorized private practices>, your opinion concerns: |
| U.67.2.6 | For <The equipment>, your opinion concerns: |  | If SERVICES |
| U.67.2.7 | For <Other>, your opinion concerns: | HP.67.2.1 | For <Their availability>, your opinion concerns: |
|  | If DRUGS AND PHARMACEUTICALS | HP.67.2.2 | For <Their accessibility>, your opinion concerns: |
| U.67.3.1 | For <Their availability at the health facility>, your opinion concerns: | HP.67.2.3 | For <Their organization>, your opinion concerns: |
| U.67.3.2 | For <The explanations given by the depot manager>, your opinion concerns: | HP.67.2.4 | For <Their quality>, your opinion concerns: |
| U.67.3.3 | For <The packaging of the drugs>, your opinion concerns: | HP.67.2.5 | For <Their effectiveness,>, your opinion concerns: |
| U.67.3.4 | For <Other>, your opinion concerns: | HP.67.2.6 | For <Other>, your opinion concerns: |
|  | If PRICE |  | If FREE CARE |
| U.67.4.1 | For <The price of the consultation>, your opinion concerns: | HP.67.9.1 | For <Pregnant women>, your opinion concerns: |
| U.67.4.2 | For <The price of the drugs>, your opinion concerns: | HP.67.9.2 | For <Children under five years old>, your opinion concerns: |
| U.67.4.3 | For <The price of tests and/or other examinations>, your opinion concerns: | HP.67.9.3 | For <Indigents>, your opinion concerns: |
| U.67.4.4 | For <Other>, your opinion concerns: |  | If HUMAN RESOURCES |
|  | If FREE CARE | HP.67.3.1 | For <Their training>, your opinion concerns: |
| U.67.5.1 | For <Pregnant women>, your opinion concerns: | HP.67.3.2 | For <Their quantity>, your opinion concerns: |
| U.67.5.2 | For <Children under 5 years old>, your opinion concerns: | HP.67.3.3 | For <Their diversity>, your opinion concerns: |
| U.67.5.3 | For <Indigents>, your opinion concerns: | HP.67.3.4 | For <Their distribution>, your opinion concerns: |
|  |  | HP.67.3.5 | For <Their management>, your opinion concerns: |
|  |  | HP.67.3.6 | For <Their remuneration>, your opinion concerns: |
|  |  | HP.67.3.7 | For <Their performance>, your opinion concerns: |
|  |  | HP.67.3.8 | For <Their career>, your opinion concerns: |
|  |  | HP.67.3.9 | For <Other>, your opinion concerns: |
|  |  |  | If DRUGS AND MEDICAL PRODUCTS |
|  |  | HP.67.4.1 | For <Their national production>, your opinion concerns: |
|  |  | HP.67.4.2 | For <Their supply>, your opinion concerns: |
|  |  | HP.67.4.3 | For <Their quality>, your opinion concerns: |
|  |  | HP.67.4.4 | For <Their availability>, your opinion concerns: |
|  |  | HP.67.4.5 | For <Their storage>, your opinion concerns: |
|  |  | HP.67.4.6 | For <Their use>, your opinion concerns: |
|  |  | HP.67.4.7 | For <Their affordability>, your opinion concerns: |
|  |  | HP.67.4.8 | For <Other>, your opinion concerns: |
|  |  |  | If INFRASTRUCTURE, EQUIPMENT & MAINTENANCE |
|  |  | HP.67.5.1 | For <Infrastructure>, your opinion concerns: |
|  |  | HP.67.5.2 | For <Equipment>, your opinion concerns: |
|  |  | HP.67.5.3 | For <Maintenance>, your opinion concerns: |
|  |  | HP.67.5.4 | For <All three>, your opinion concerns: |
|  |  |  | If HEALTH INFORMATION SYSTEMS |
|  |  | HP.67.6.1 | For <Data quality>, your opinion concerns: |
|  |  | HP.67.6.2 | For <Validity of the results produced>, your opinion concerns: |
|  |  | HP.67.6.3 | For <Results relevance>, your opinion concerns: |
|  |  | HP.67.6.4 | For <Results use>, your opinion concerns: |
|  |  | HP.67.6.5 | For <Other>, your opinion concerns: |
|  |  |  | If RESEARCH |
|  |  | HP.67.7.1 | For <The relevance of the research to the country>, your opinion concerns: |
|  |  | HP.67.7.2 | For <Validity of the research results>, your opinion concerns: |
|  |  | HP.67.7.3 | For <Accessibility of research results>, your opinion concerns: |
|  |  | HP.67.7.4 | For <Diffusion to decision-makers>, your opinion concerns: |
|  |  | HP.67.7.5 | For <Use of results by decision-makers>, your opinion concerns: |
|  |  | HP.67.7.6 | For < Research financing >, your opinion concerns: |
|  |  |  | If FINANCING |
|  |  | HP.67.8.1 | For <Health facility budgets>, your opinion concerns: |
|  |  | HP.67.8.2 | For <Fee-for-service patient care>, your opinion concerns: |
|  |  | HP.67.8.3 | For <Free care for indigents>, your opinion concerns: |
|  |  | HP.67.8.4 | For <Grant programs>, your opinion concerns: |
|  |  | HP.67.8.5 | For <Mutual health insurance>, your opinion concerns: |
|  |  | HP.67.8.6 | For <Other>, your opinion concerns: |
|  |  |  |  |
