# Supplementary material for: Using Information and Communication Technologies to Engage Citizens in Health System Governance in Burkina Faso: Protocol for Action Research
Source: JMIR Res Protoc. 2021 Nov 16;10(11):e28780. doi: 10.2196/28780 (PMC8663653; doi:10.2196/28780)
Supplement: Multimedia Appendix 4 [file resprot_v10i11e28780_app4.docx]

**S4 Appendix: Interview guide for evaluation of the deliberative workshop**

**INTERVIEW GUIDE**

*Evaluation of the deliberative workshop*

“Your participation in this interview is requested to evaluate the organization, the conduct, and the potential impacts of the deliberative workshop held last (date), where the research results of the TOPICs project were presented and discussed.

The objective in this interview is to engage participants in a critical reflection on how the workshop could be improved. The goal is to make it more beneficial and useful for all those affected by the issue of health care quality.

Your experience will help strengthen future knowledge transfer and sharing efforts in Burkina Faso. So, in this consultation, I’m going to ask you a few questions on different themes surrounding the workshop.”

- First, here are some clarifications regarding the confidentiality of the information provided during this interview and anonymity.
- The main objective of this interview is to take stock of the workshop's usefulness for your professional practice and for population health generally.
- Any knowledge provided will be kept confidential by the interviewers.
- All information provided will be treated and presented anonymously.
- At any time, you may withdraw from the workshop without any explanation.
- Do you agree to have the interview digitally recorded?

**Context setting**

Do you have any questions before we get started?

1. What is your current position?

2. Could you briefly describe your mandate within your organization?

4. How would you depict the attitudes and perceptions of those responsible for the quality of care (including different organizations, associations, and decision makers) concerning research generally?

… and the activities of researchers (or evaluators) specifically?

**Assessment of the workshop’s proceedings and content**

5. Did you attend at all the activities of the day?

6. To begin, what is your assessment of the deliberative workshop of last (date)?

7. During the morning, research results were presented.

— Did the researchers use proper and accessible language?

— Were the presentation formats appropriate (legible, content, etc.)?

— What did you discover most from these presentations? Or what did you learn from the day?

6. How did the afternoon’s breakout sessions go for you?

7. What did you think of the large group's return at the end of the day? [Refresh memory if necessary]

8. What do you think of setting up a committee to follow up on the recommendations from the workshop that were proposed at the end of the day?

9. What did you think of the group dynamics among the participants?

— In terms of the composition of the group, do you think it was a good thing to invite actors from different backgrounds?

**Appreciation of the research notes**

10. Did you review the research notes provided to participants before the workshop (thoroughly, partially, not at all)?

— If so, what is your assessment? [show them copies of the notes]

— Was there enough information, or was there unnecessary information?

— If not, why didn’t you read it?

— Do you have any suggestions on how to improve these ratings (or their impact)?

**The usefulness of knowledge & impact of the workshop**

11. How might the results produced by the researchers be useful?

— In concrete terms, how could these results be useful for your practice? [ask for examples]

— Has anything changed in your practice since this workshop? Or have you personally taken any action as a result of the workshop?

12. What is the impact of such a workshop in the short and medium-term, in your opinion?

13. What are the obstacles to using the results presented during this workshop?

14. What more could be done to improve the use of the knowledge produced by the researchers?

15. How can workshop participants become bearers of the knowledge transmitted and discussed during the day?

**Involvement of decision-makers**

16. What would be the best way to foster the engagement of political actors in such knowledge transfer activities?

17. In your opinion, how could collaboration between researchers and policy actors, those in a position to make decisions, be fostered?

**Others**

18. Can you think of any other ways in which the research results of this project could be made more widely known and useful?

I’ve asked the gist of my questions. Is there anything else you would like to address?

**Questionnaire**

General information from participants

1. **What gender are you?**

| Male |  |
| --- | --- |
| Female |  |

**2. Indicate your age in years:_______________.**

**3. What is the last degree you obtained ?**

| Elementary School |  |
| --- | --- |
| Middle School |  |
| High School Diploma |  |
| Undergraduate 2 – Associate Degree |  |
| Bachelor’s |  |
| Postgraduate 1 (Master’s 1) |  |
| Postgraduate 2 (Master’s 2) |  |
| PhD |  |

**4. What job/position do you hold?**

___________________________________________________________________________.

**5. How long have you been working in the field of health (health association, decision-makers, insurers, physicians, health worker, nurse manager, district health office social care, social work, National Health Insurance Fund (or in a project that affects population health)?**

**Enter the number of years: ______________**
