# Supplementary material for: Using Information and Communication Technologies to Engage Citizens in Health System Governance in Burkina Faso: Protocol for Action Research
Source: JMIR Res Protoc. 2021 Nov 16;10(11):e28780. doi: 10.2196/28780 (PMC8663653; doi:10.2196/28780)
Supplement: Multimedia Appendix 5 [file resprot_v10i11e28780_app5.docx]

**S5 Appendix: Workshop Evaluation QUESTIONNAIRE**

Workshop Evaluation QUESTIONNAIRE

All information provided will be kept strictly confidential, and your responses will be forwarded only to the researchers on the workshop evaluation team. Your participation is entirely voluntary, and you are free to withdraw at any time. However, your opinion and suggestions are critical in understanding how useful the workshop was and how we might improve subsequent workshops. We also want to know if the discussions at the workshop have enabled or will enable the use of research knowledge that was shared and discussed during the day.

We would greatly appreciate it if you would take the time to complete this short questionnaire at the end of the day. Please return it to the investigators responsible for evaluating the workshop afterward.

If you have any inquiries about your rights as a participant in this study, please contact Christian Dagenais, Professor in the Department of Psychology at the Université de Montréal by email at christian.dagenais@umontreal.ca.

**SECTION 1- General Information**

1. **What gender are you? (Check the appropriate box)**

| Male |  |
| --- | --- |
| Female |  |

**2. Indicate your age in years:_______________.**

**3. What is the last degree you obtained? (Check the appropriate box)**

| Elementary School |  |
| --- | --- |
| Middle School |  |
| High School Diploma |  |
| Undergraduate 2 – Associate Degree |  |
| Bachelor’s |  |
| Postgraduate 1 (Master’s 1) |  |
| Postgraduate 2 (Master’s 2) |  |
| PhD |  |

**4. What job/position do you hold?**

___________________________________________________________________________.

**5. How long have you been working in the field of health (health association, decision-makers, insurers, physicians, health worker, nurse manager, district health office social care, social work, National Health Insurance Fund (or in a project that affects population health)?**

**Enter the number of years: ______________**

**SECTION 2—Reactions from workshop participants**

| 1. What were your expectations when you decided to participate in this activity? |
| --- |
| \| **For each of the following statements, circle the number in the right-hand column that corresponds to your agreement level.** \| \| --- \|  \| Strongly disagree \| Disagree \| Somewhat disagree \| Neither agree nor disagree \| Somewhat agree \| Agree \| Strongly agree \| \| --- \| --- \| --- \| --- \| --- \| --- \| --- \| \| 1 \| 2 \| 3 \| 4 \| 5 \| 6 \| 7 \| |

| **Workshop objectives and content** | |
| --- | --- |
| 2. The content of this workshop met my expectations | 1 2 3 4 5 6 7 |
| 3. I'm satisfied with the following: | |
| 3.1 The topics covered | 1 2 3 4 5 6 7 |
| 3.2 The quality of the debates | 1 2 3 4 5 6 7 |
| 3.3 The quality of the presentations | 1 2 3 4 5 6 7 |
| 4. The workshop was new to me | 1 2 3 4 5 6 7 |
| 5. The content presented was understandable | 1 2 3 4 5 6 7 |
| 6. The information submitted will be useful to me in my work | 1 2 3 4 5 6 7 |
|  |  |
| **Workshop organization** | |
| 1. I am satisfied with the following: | |
| 7.1 The quality of the documents distributed | 1 2 3 4 5 6 7 |
| 7.2 The presentation medium | 1 2 3 4 5 6 7 |
| 7.3 The meeting room | 1 2 3 4 5 6 7 |
| 7.4 The meal breaks | 1 2 3 4 5 6 7 |

| **General appreciation and suggestions for improvement** |
| --- |

1. What did you most appreciate about the workshops? ____________________________________________________________________________________________________________________________________________________________________________________________________________________________________________________________________________________________________________________________
2. What did you like least about these workshops?

____________________________________________________________________________________________________________________________________________________________________________________________________________________________________________________________________________________________________________________________

1. What are your suggestions (content, materials, other) to improve these workshops?

____________________________________________________________________________________________________________________________________________________________________________________________________________________________________________________________________________________________________________________________

**SECTION 3- Intent to use knowledge questionnaire (adapted from Boyko et al., 2011** [63]**)**

| \| **For each of the following statements, circle the number in the right-hand column that corresponds to your level of agreement** \| \| --- \| |
| --- | --- |

| Strongly disagree | Disagree | Somewhat disagree | Neither agree nor disagree | Somewhat agree | Agree | Strongly agree |
| --- | --- | --- | --- | --- | --- | --- |
| 1 | 2 | 3 | 4 | 5 | 6 | 7 |

| 1. I expect to use the (some of the) research data that was mentioned at the workshop to help me in my work. | 1 2 3 4 5 6 7 | |
| --- | --- | --- |
| 1. I intend to use the research data (or part of it) discussed at the workshop to help me in my work. | 1 2 3 4 5 6 7 | |
| 1. I already see an opportunity where I can use the research data (or part of it) referred to in the workshop to help me in my work. | 1 2 3 4 5 6 7 | |
| 1. I'm expected to use research data of the type that was presented at the workshop. | 1 2 3 4 5 6 7 | |
| 1. I feel social or political pressure to use research data of the type that was presented at the workshop. | 1 2 3 4 5 6 7 | |
| 1. Most people who are important to me in my professional life think that I should use research data of the type that was presented at the workshop. | 1 2 3 4 5 6 7 | |
| 1. I'm confident that I could use research data of the type presented at the workshop. | 1 2 3 4 5 6 7 | |
| 1. The decision to use research data of the type presented at the workshop is out of my control. | 1 2 3 4 5 6 7 | |
| 1. The decision to use research data of the type presented at the workshop is solely up to me. | 1 2 3 4 5 6 7 | |
| **For each of the following statements, circle the number that corresponds to your opinion**. | |  |

10a. The use of research evidence of the type presented at the workshop is....

| Very harmful | Moderately  harmful | Slightly harmful | Neutral | Slightly beneficial | Moderately beneficial | Very beneficial |
| --- | --- | --- | --- | --- | --- | --- |
| 1 | 2 | 3 | 4 | 5 | 6 | 7 |

10b. The use of research evidence of the type presented at the workshop is....

| Very bad | Moderately bad | Slightly bad | Neutral | Slightly good | Moderately good | Very good |
| --- | --- | --- | --- | --- | --- | --- |
| 1 | 2 | 3 | 4 | 5 | 6 | 7 |

10c. The use of research evidence of the type presented at the workshop is....

| Very unpleasant  (for me) | Moderately  unpleasant (for me) | Slightly  unpleasant  (for me) | Neutral | Slightly pleasant (for me) | Moderately pleasant (for me) | Very pleasant (for me) |
| --- | --- | --- | --- | --- | --- | --- |
| 1 | 2 | 3 | 4 | 5 | 6 | 7 |

10d. The use of research evidence of the type presented at the workshop is....

| Very unhelpful | Moderately  unhelpful | Slightly unhelpful | Neutral | Slightly helpful | Moderately helpful | Very helpful |
| --- | --- | --- | --- | --- | --- | --- |
| 1 | 2 | 3 | 4 | 5 | 6 | 7 |

11. Regarding my use of research evidence of the type that was presented at the workshop, most people who are important to me in my professional life think that…

| I should definitely not | I should almost certainly not | I should probably not | Neutral | I should probably | I should almost certainly | I should definitely |
| --- | --- | --- | --- | --- | --- | --- |
| 1 | 2 | 3 | 4 | 5 | 6 | 7 |

11. For me to use research evidence of the type that was discussed at the workshop is…

| Very difficult | Moderately difficult | Slightly difficult | Neutral | Slightly easy | Moderately easy | Very easy |
| --- | --- | --- | --- | --- | --- | --- |
| 1 | 2 | 3 | 4 | 5 | 6 | 7 |

**Additional file 4: Interview guide for evaluation of the deliberative workshop**

**INTERVIEW GUIDE**

*Evaluation of the deliberative workshop*

“Your participation in this interview is requested to evaluate the organization, the conduct, and the potential impacts of the deliberative workshop held last (date), where the research results of the TOPICs project were presented and discussed.

The objective in this interview is to engage participants in a critical reflection on how the workshop could be improved. The goal is to make it more beneficial and useful for all those affected by the issue of health care quality.

Your experience will help strengthen future knowledge transfer and sharing efforts in Burkina Faso. So, in this consultation, I’m going to ask you a few questions on different themes surrounding the workshop.”

- First, here are some clarifications regarding the confidentiality of the information provided during this interview and anonymity.
- The main objective of this interview is to take stock of the workshop's usefulness for your professional practice and for population health generally.
- Any knowledge provided will be kept confidential by the interviewers.
- All information provided will be treated and presented anonymously.
- At any time, you may withdraw from the workshop without any explanation.
- Do you agree to have the interview digitally recorded?

**Context setting**

Do you have any questions before we get started?

1. What is your current position?

2. Could you briefly describe your mandate within your organization?

4. How would you depict the attitudes and perceptions of those responsible for the quality of care (including different organizations, associations, and decision makers) concerning research generally?

… and the activities of researchers (or evaluators) specifically?

**Assessment of the workshop’s proceedings and content**

5. Did you attend at all the activities of the day?

6. To begin, what is your assessment of the deliberative workshop of last (date)?

7. During the morning, research results were presented.

— Did the researchers use proper and accessible language?

— Were the presentation formats appropriate (legible, content, etc.)?

— What did you discover most from these presentations? Or what did you learn from the day?

6. How did the afternoon’s breakout sessions go for you?

7. What did you think of the large group's return at the end of the day? [Refresh memory if necessary]

8. What do you think of setting up a committee to follow up on the recommendations from the workshop that were proposed at the end of the day?

9. What did you think of the group dynamics among the participants?

— In terms of the composition of the group, do you think it was a good thing to invite actors from different backgrounds?

**Appreciation of the research notes**

10. Did you review the research notes provided to participants before the workshop (thoroughly, partially, not at all)?

— If so, what is your assessment? [show them copies of the notes]

— Was there enough information, or was there unnecessary information?

— If not, why didn’t you read it?

— Do you have any suggestions on how to improve these ratings (or their impact)?

**The usefulness of knowledge & impact of the workshop**

11. How might the results produced by the researchers be useful?

— In concrete terms, how could these results be useful for your practice? [ask for examples]

— Has anything changed in your practice since this workshop? Or have you personally taken any action as a result of the workshop?

12. What is the impact of such a workshop in the short and medium-term, in your opinion?

13. What are the obstacles to using the results presented during this workshop?

14. What more could be done to improve the use of the knowledge produced by the researchers?

15. How can workshop participants become bearers of the knowledge transmitted and discussed during the day?

**Involvement of decision-makers**

16. What would be the best way to foster the engagement of political actors in such knowledge transfer activities?

17. In your opinion, how could collaboration between researchers and policy actors, those in a position to make decisions, be fostered?

**Others**

18. Can you think of any other ways in which the research results of this project could be made more widely known and useful?

I’ve asked the gist of my questions. Is there anything else you would like to address?

**Questionnaire**

General information from participants

1. **What gender are you?**

| Male |  |
| --- | --- |
| Female |  |

**2. Indicate your age in years:_______________.**

**3. What is the last degree you obtained ?**

| Elementary School |  |
| --- | --- |
| Middle School |  |
| High School Diploma |  |
| Undergraduate 2 – Associate Degree |  |
| Bachelor’s |  |
| Postgraduate 1 (Master’s 1) |  |
| Postgraduate 2 (Master’s 2) |  |
| PhD |  |

**4. What job/position do you hold?**

___________________________________________________________________________.

**5. How long have you been working in the field of health (health association, decision-makers, insurers, physicians, health worker, nurse manager, district health office social care, social work, National Health Insurance Fund (or in a project that affects population health)?**

**Enter the number of years: ______________**
